# Plastic Responses of a Sessile Prey to Multiple Predators: A Field and Experimental Study

**DOI:** 10.1371/journal.pone.0115192

**Published:** 2014-12-17

**Authors:** Philipp Emanuel Hirsch, David Cayon, Richard Svanbäck

**Affiliations:** Department of Ecology and Evolution, Limnology, Evolutionary Biology Centre, Uppsala University, Uppsala, Sweden; University of Calgary, Canada

## Abstract

**Background:**

Theory predicts that prey facing a combination of predators with different feeding modes have two options: to express a response against the feeding mode of the most dangerous predator, or to express an intermediate response. Intermediate phenotypes protect equally well against several feeding modes, rather than providing specific protection against a single predator. Anti-predator traits that protect against a common feeding mode displayed by all predators should be expressed regardless of predator combination, as there is no need for trade-offs.

**Principal Findings:**

We studied phenotypic anti-predator responses of zebra mussels to predation threat from a handling-time-limited (crayfish) and a gape-size-limited (roach) predator. Both predators dislodge mussels from the substrate but diverge in their further feeding modes. Mussels increased expression of a non-specific defense trait (attachment strength) against all combinations of predators relative to a control. In response to roach alone, mussels showed a tendency to develop a weaker and more elongated shell. In response to crayfish, mussels developed a harder and rounder shell. When exposed to either a combination of predators or no predator, mussels developed an intermediate phenotype. Mussel growth rate was positively correlated with an elongated weaker shell and negatively correlated with a round strong shell, indicating a trade-off between anti-predator responses. Field observations of prey phenotypes revealed the presence of both anti-predator phenotypes and the trade-off with growth, but intra-specific population density and bottom substrate had a greater influence than predator density.

**Conclusions:**

Our results show that two different predators can exert both functionally equivalent and inverse selection pressures on a single prey. Our field study suggests that abiotic factors and prey population density should be considered when attempting to explain phenotypic diversity in the wild.

## Introduction

Phenotypic plasticity is a common response of organisms to environmental variation and an important source of diversity within and across populations [1]. Amongst the best-studied examples of phenotypic plasticity are anti-predator responses and predation is believed to be one of the most important factors influencing phenotypes in natural populations [2], [3]. In nature, predation rarely results from a single predator but from a community including a multitude of predators [4]–[6], each with different feeding modes that can lead to opposing or functionally inverse selection pressures on prey phenotypes [5], [7]. Thus, a decisive component of the feeding mode is characteristic to one predator but absent or irrelevant in the other. For example, in aquatic ecosystems fish predators are typically gape-size-limited: they are unable to consume prey greater than the width of their oral cavity. In contrast, many invertebrate predators can consume prey much larger than themselves and are limited by the abilities of their external feeding apparatus to capture and handle prey [8].

**Figure 1:**
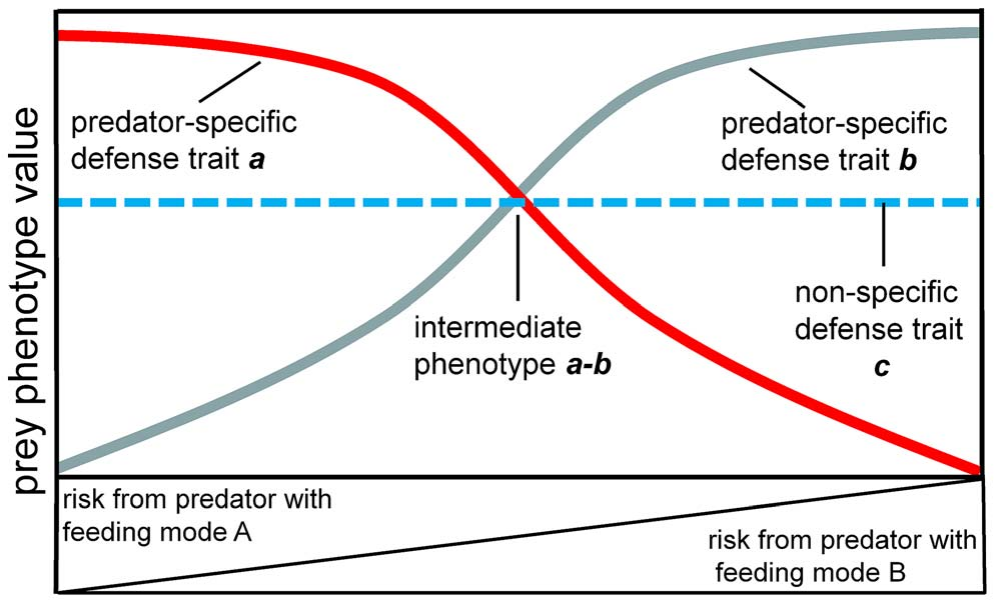
Conceptual model of prey responses to multiple predators. The figure depicts a prey responding with different traits to two predator types that are functionally equivalent in their initial step of prey acquisition (i.e. identical feeding mode C) and functionally inverse in their further feeding modes (i.e. opposing feeding modes A and B). At any combination of density of predators the prey expresses the non-specific defense trait, *c*, at the same level simply because it perceives cues for predation risk from any of the predators that share feeding mode C. As the risk from the predator with feeding mode A increases, the prey expresses a specific defense trait, *a*, that optimally protects against that single predator. For instance, assume that the concentration of cues in the form of kairomones or alarm cues from conspecifics being consumed by a certain predator type increases, then the prey develops certain phenotypic defense traits to avoid consumption by that specific predator. The same holds for the trait protecting against the predator with feeding mode B: the prey expresses a specific defense phenotype, *b*, when perceiving increased predation risk from that single predator. When the prey perceives the risk from both predator types as equal, then theory predicts it should express an intermediate phenotype, *a-b*. The intermediate phenotype describes a defense that lies between the extreme ends of the phenotypes expressed in response to only a single predator i.e. a phenotype that provides equally bad or good protection against either predator type.

Based on numerous experimental studies, we now possess a conceptual understanding of how different types of predators influence plastic responses of prey [5], [9]–[11]. Consider a situation with two predators and a prey species. In the complete absence of predators the prey species produces a phenotype, *x*, that maximizes the energy reserves it can use towards growth and reproduction. However, predation shifts energy expenditure towards anti-predator responses, inducing specific anti-predator phenotypes. If both predators are equivalent in their first step of prey acquisition, feeding mode *C*, this is countered in the prey by phenotypic response *c*. Ecological theory predicts that expression of a universal anti-predator response, such as *c*, should not depend on which of two functionally equivalent predators poses a risk ([11], Fig. 1). Now consider that the two predators are functionally inverse in their further feeding modes: predator 1 uses feeding mode *A* and predator 2 feeding mode *B*, which are countered in the prey with phenotypic responses *a* and *b* respectively. Both phenotypic responses *a* and *b* cannot be maximally expressed due to trade-offs, so in the presence of both predators an intermediate response, *a-b*, should be observed. If risks from the two predator types are comparable, then this intermediate trait should provide equal protection from them both ([9], [11] Fig. 1)

The conceptual differences in anti-predator trait expression (Fig. 1) result from trade-offs. Anti-predator responses entail energy costs for the modulation of the phenotype. These costs have to be weighed against the benefits of predator protection. One recurrent trade-off connected to the plastic response against predators is the growth-predation risk trade-off [12], [13]. This trade-off occurs when feeding modes *A* and *B* represent differences in gape-size-limited vs. handling-time-limited feeding modes. In this case, fast growth increases protection from predators that are gape-size-limited, but frequently makes the prey more susceptible to gape-unconstrained predators [12], [13]. For example, in anurans the attainment of a size refuge from gape-size-limited predators requires increased foraging movement, which increases the risk of encountering gape-unconstrained sit-and-wait predators [12]–[15].

Compared to our growing experimental understanding of prey responses to multiple predators, we still know little about how anti-predator phenotypes are expressed in the field [9], [16]. A variety of environmental stimuli potentially affect phenotype expression in the wild [16], [17]. Therefore, to understand if and how predation acts to shape phenotypic differences in the field, we need to address how other abiotic or biotic factors influence phenotypic expression [18]. For example, anti-predator responses in the shell shape of winkles (*Littorina* spp.) serve as a textbook example for experimentally inducible phenotypes. Yet in the field abiotic factors such as wave exposure and temperature also affect shell phenotypes [19], [20].

In the present study we investigated plastic anti-predator responses of a sessile prey, the zebra mussel (*Dreissena polymorpha*), to roach (*Rutilus rutilus*) and signal crayfish (*Pacifastacus leniusculus*) predators. The two predators are functionally equivalent in their initial step of prey acquisition, i.e. predators 1 and 2 share an initial feeding mode *C*, but are functionally inverse in the way they consume the prey, i.e. predators 1 and 2 have opposing further feeding modes, *A* and *B*. Prior to prey consumption, both roach and crayfish have to dislodge mussels from the substrate. Mussels avoid being removed from the substrate by excreting byssus threads that firmly attach to surfaces, i.e. phenotypic response *c*. After having dislodged the mussel the further feeding mode is functionally inverse in roach and crayfish. Roach will swallow a mussel whole and then process it through the buccal cavity. Hence, each mussel needs to pass through the gape before it is later ground by the pharyngeal teeth. Previous experiments found the gape size and width to be the limiting factor for mussel consumption in roach [21]. In contrast, crayfish have to handle the mussel with their first and second pair of pleopods. They turn the mussel around until the sharp edge of the shell is optimally positioned for crushing with the mandibles [22], [23]. The similar first step of prey consumption and the opposing feeding modes of both predators suggest that zebra mussels, roach and crayfish are suitable study organisms to test our aforementioned conceptual model (Fig. 1).

The model predicts that non-specific anti-predator traits will be equally expressed and not entail any trade-offs in predator susceptibility. Energetic trade-offs of non-specific traits might be present because they require tissue synthesis. But the expression of non-specific defenses against one predator, even if energetically costly, will not increase susceptibility to any other predator types. Predator-specific responses will lead to a growth-predation risk trade-off between protection against one and susceptibility to another predator. For a gape-size-limited predator like roach the prey size is important. To avoid ingestion by roach the mussel should try to grow fast to quickly become greater than the predator's gape (phenotypic response *a*). On the other hand, developing a harder, rounder shell that better resists efficient handling and crushing forces would protect from handling-time-limited crayfish predators (phenotypic response *b*). However, while providing protection from crushing predators, the hardening of the shell might be incompatible with fast growth. This is because shell calcification is rate- rather than energy-limited, i.e. a mussel can only deposit a fixed amount of calcium per time in its shell [24]. With the rate of shell hardening being fixed the mussels cannot achieve a faster shell-calcification by investing a larger fraction of their energy budget into the calcification process. Thus, mussels cannot achieve both a fast growth rate and a fast shell hardening rate [24]–[26].

Therefore, based on the model and the characteristics of our study organisms we make the following predictions: *i*) Mussels will increase attachment strength equally, as an unspecific anti-predator response, when exposed to both predator types alone or combined. *ii*) Mussels will grow faster and develop a large and weak shell in response to the gape-size-limited roach, whereas the handling-time-limited crayfish should elicit slower growth and the development of a harder and rounder shell. *iii*) When exposed to a combination of both predators, mussels will express an intermediate phenotype. *iv*) When not exposed to predators, anti-predator responses should be absent, i.e. byssus threads should be expressed at the minimum necessary for attachment, shell strength and shell shape should reflect a phenotype optimized for growth and reproduction.

To test these predictions we investigated the phenotypic responses of mussels to roach and crayfish, and the associated trade-offs, under experimental conditions. We then tested our experimental predictions in a field survey by relating mussel phenotypes to local densities of roach and crayfish at different sites within a lake and testing for the presence of trade-offs between growth and shell strength. We expected that predator density would affect phenotype expression in a fashion similar to that seen in the experiment. Finally, we also assessed a large range of additional biotic and abiotic factors in the field, expecting to find other factors relevant for phenotype expression in zebra mussels.

## Results

### Experiment

**Figure 2:**
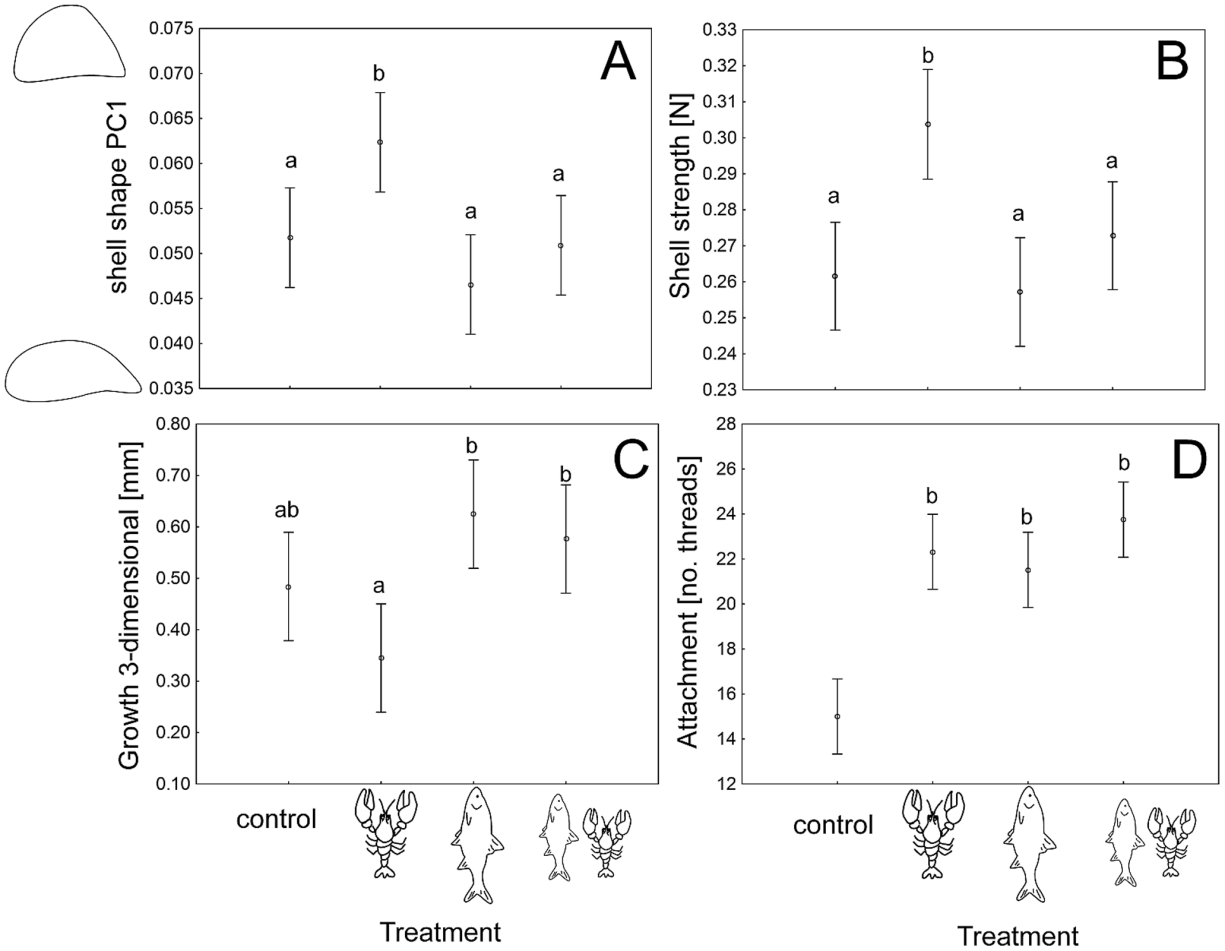
Zebra mussels' phenotype expression changes with predation risk. Anti-predator responses of zebra mussels from the four experimental treatments: either a single crayfish (crayfish symbol), a single roach (fish symbol), both predators combined (both symbols next to each other) or no predator at all (minus symbol). Different lower case a and b letters denote significant differences for p<0.05 after post-hoc tests. Error bars denote 95% confidence intervals **A**: shell shape expressed as a principal component PC1 explaining 77% of the total variation in the contour shape of all analyzed mussels (the contours on the y-axis depict ±2SD of the mean of PC1). **B**: shell strength as resistance to crushing in the form of the residuals from the correlation between mussel size and strength. **C**: growth measured in mm increase in the three shell dimensions length, height, and width compared to the start of experiment. **D**: Attachment strength as inferred from the number of byssus threads with which individual zebra mussels were attached to the surface.

In the experiment, mussels showed phenotypic differences in response to single, combined and no-predator treatments. As predicted from our model (Fig. 1) the attachment strength (approximated as the number of byssus threads of mussels) increased in all the predator treatments compared to the control (Nested ANOVA, F_3,420_ = 23.98, p<0.001, Fig. 2D). The mussel phenotypes differed significantly between treatments: when exposed to crayfish mussels developed a rounder shell. Exposure to roach appeared to result in a more elongated shell, although shell shape in response to roach was not significantly different from the combined or control treatments (Nested ANCOVA, response variable PC1 describing 63% of the variation in shape, F_3,438.1_ = 3.95, p = 0.008, Fig. 2A). The shell strength (measured as resistance to a crushing force in Newton) also showed predator-specific differences: mussels exposed to crayfish had the strongest shell while exposure to roach tended to result in a weaker shell (Nested ANCOVA, F_3,442.9_ = 7.81, p<0.001, Fig. 2B). However, neither shell shape nor strength differed significantly between the combined, the control and the roach treatment (Fig. 2A, B). Shell shape measured as PC2 (describing 9% of the variation in shell shape) did not differ between treatments (Nested ANCOVA, F_3,437.6_ = 1.87, p = 0.13).

**Figure 3:**
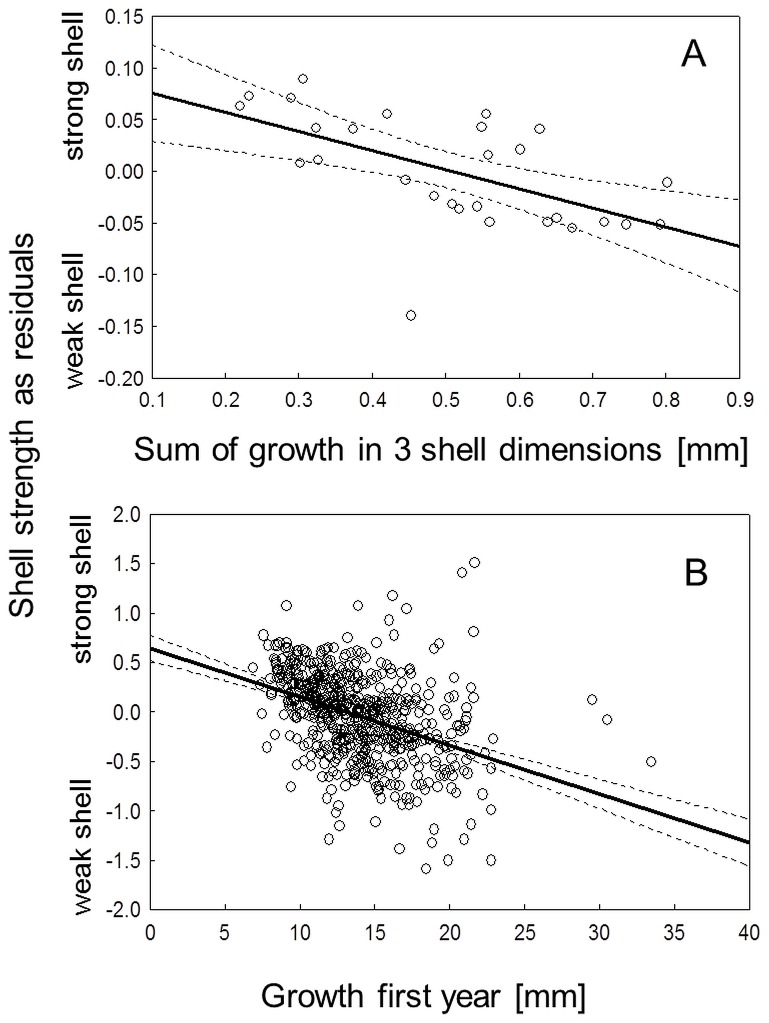
Shell strength and growth are correlated in zebra mussels. **A**: experimental data as mean growth (measured in mm increase in the 3 shell dimensions length, height, and width) in relation to the corresponding shell strength means from the 28 experimental tanks with 16 mussels each. **B**: field data as individual growth in the first year post settlement in relation to shell strength of 556 mussels. Dashed lines denote 95% confidence intervals for the fit.

**Figure 4:**
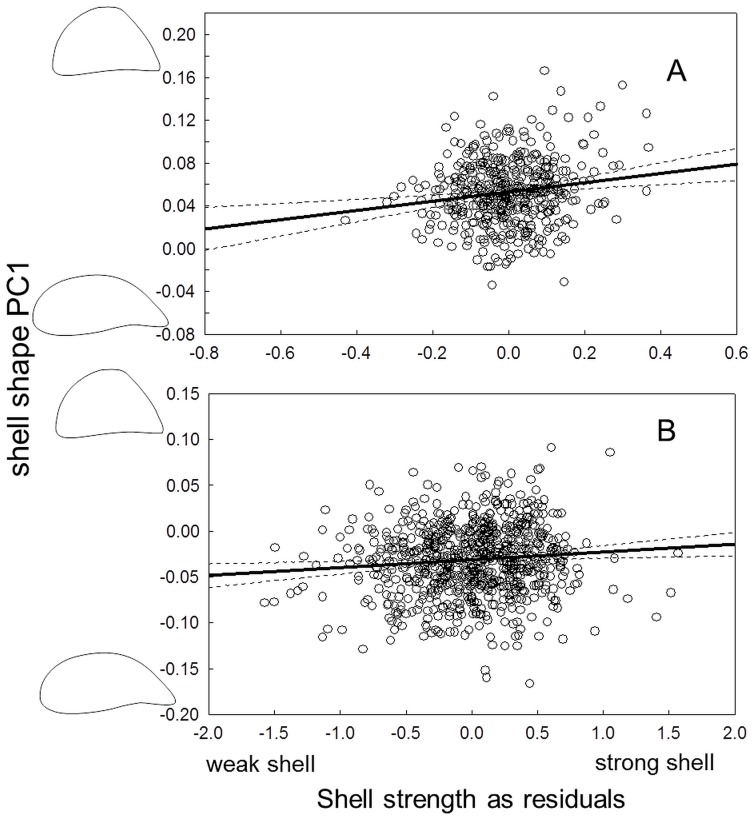
Shell shape and shell strength are correlated in zebra mussels. **A**: experimental data from 448 mussels. **B**: field data from 756 mussels. The contours on the y-axis depict ±2SD of the mean of PC1. Dashed lines denote 95% confidence intervals for the fit. Note that we here present individual values for the experimental mussels since we obtained both a shape and strength measure for each mussel separately.

**Figure 5:**
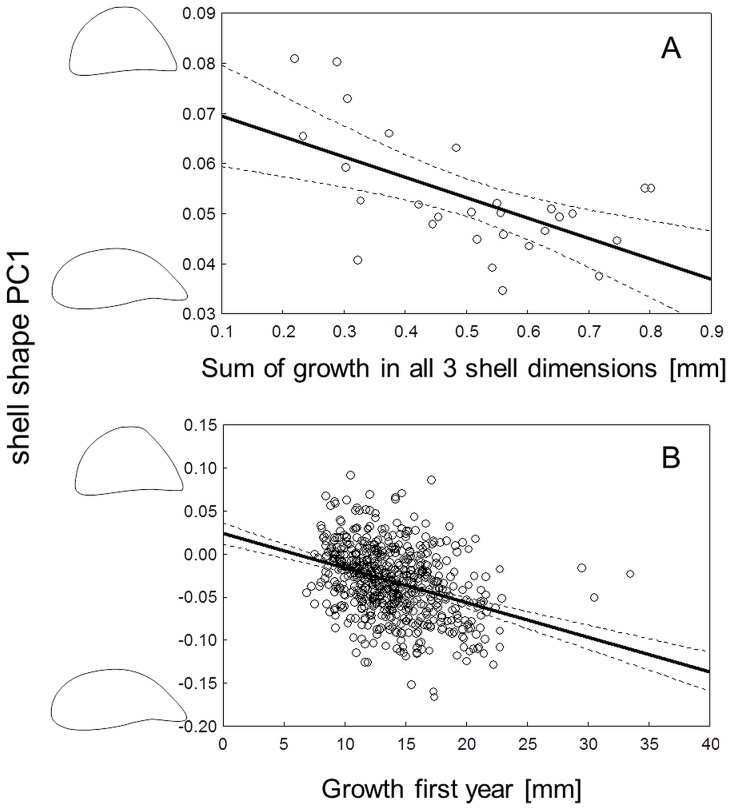
Shell shape and growth are correlated in zebra mussels. **A**: experimental data as mean growth (measured in mm increase in the 3 shell dimensions length, height, and width) in relation to the corresponding shell shape as means from the 28 experimental tanks with 16 mussels each. **B**: field data as individual growth in the first year post settlement in relation to individual shape of 556 mussels. The contours on the y-axis depict ±2SD of the mean of PC1. Dashed lines denote 95% confidence intervals for the fit.

The experimental results also revealed the existence of trade-offs between different anti-predator phenotypes. Growth and strength were negatively correlated (r = −0.57, p<0.01, Fig. 3A), and rounder shells were stronger while elongated shells were weaker (r = 0.49, p<0.01, Fig. 4A). Thus, faster growth leads to not only a weaker but also to a more elongated shell, as shown by the correlation between growth and shape (r = −0.58, p<0.01, Fig. 5A). Exposure to crayfish resulted in reduced growth (measured as increase in length, width and height of the shell) compared to the combined and especially the roach treatment, in which mussels showed the highest growth (ANOVA, F_3,23_ = 5.78, p<0.01, Fig. 2C). However compared to the control, mussels in the crayfish treatment showed only a non-significant tendency to grow less.

### Field survey

**Figure 6:**
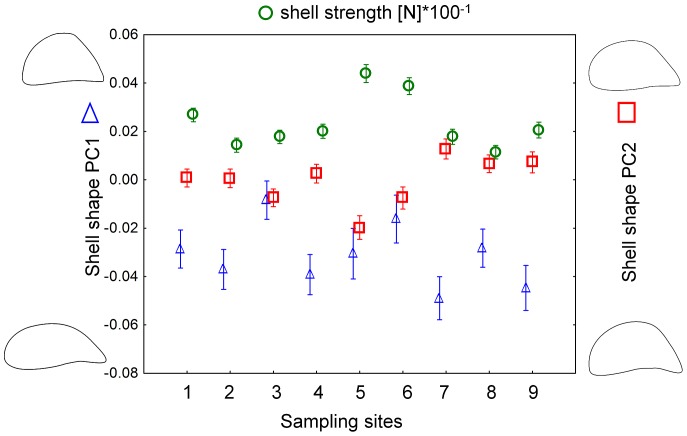
Field data reveal high phenotypic diversity in zebra mussels. Mean (±SE) shell shape (triangles = PC1, squares = PC2) and shell strength (filled circles) of 84 mussels from each of 9 sampling sites. The contours on both y-axes depict ±2SD of the mean of PC1 and PC2.

In the field, mussels showed pronounced variation in shell shape and strength among the sampling sites within the lake (Fig. 6; see Materials and Methods section for more information on the lake and sampling). Like the mussels in the experiment, mussels in the field showed signs of trade-offs between phenotypic traits and growth. Shell shape and strength were correlated: mussels with a more elongated shell were weaker than round individuals. Despite being highly significant, this relationship was very weak with an r of only 0.1 (PC1, r = 0.1, p<0.001, Fig. 4B). Mussels that grew faster in their first year post-settlement developed a more elongated shell shape; whilst mussels with lower growth built a rounder shell shape (PC1: r = −0.34, p<0.001, Fig. 5B). Furthermore, we found shell strength to be negatively correlated with growth rate also in the field (r = −0.39, p<0.001, Fig. 3B).

**Figure 7:**
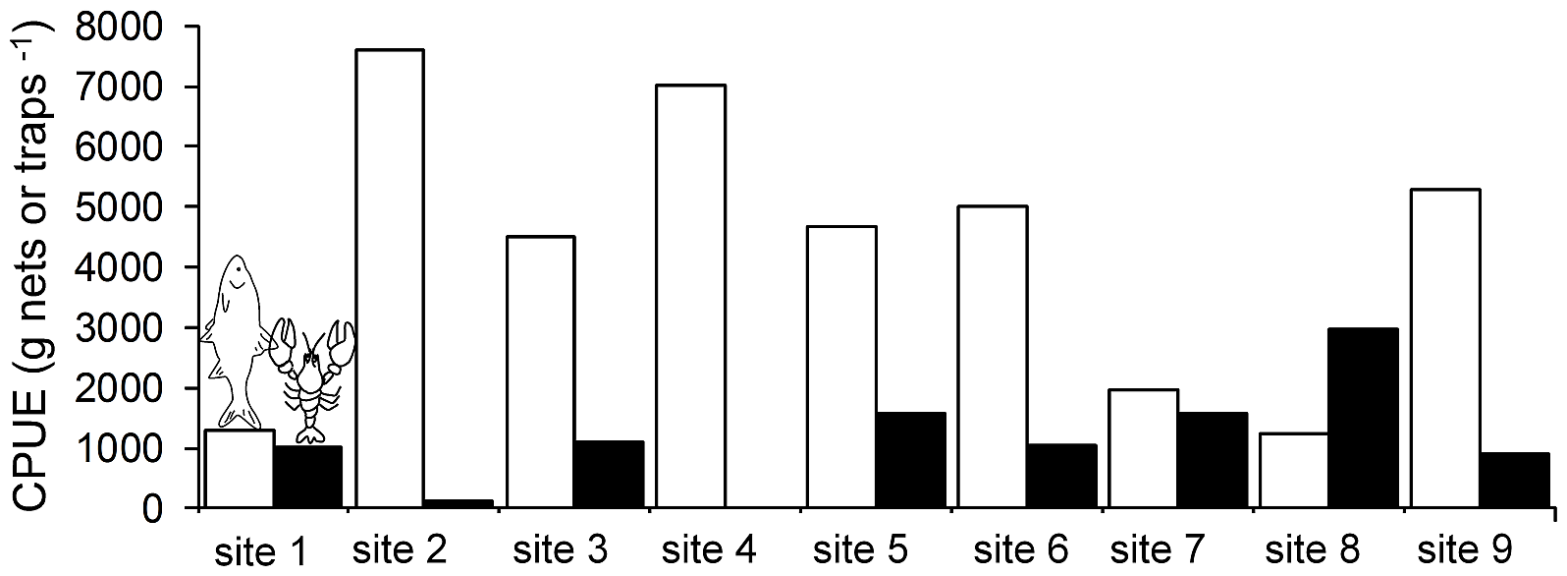
Predators of zebra mussels differ locally in their density. Density of roach (white bars, fish symbol) and crayfish (black bars, crayfish symbol) at the sampled sites expressed as catch per unit effort (four Nordic gill-nets in the case of roach and 20 baited traps in the case of crayfish). Note that crayfish from site 4 were lost after catch.

Roach, crayfish and zebra mussel densities differed substantially across the sampling sites within the lake (Fig. 7). However, the local density of crayfish and roach predators did not show significant relationships with any of the anti-predator defenses of mussels. Thus, two PLS models were constructed to explore which other parameters correlate with mussel shell phenotypes. The PLS model for shell shape had a RY of 0.36 and a Q2 of −0.01 and identified bottom structure (percent of bottom covered by plants (VIP value = 1.85) and percent of bottom covered by stones (VIP value = 1.3)), and mussel population density (VIP value = 1. 49) as the most important factors (S1 Figure). However, the negative Q2 value of the PLS model indicated that no predictor variable significantly influenced the response variables (PC1 and PC2).

**Figure 8:**
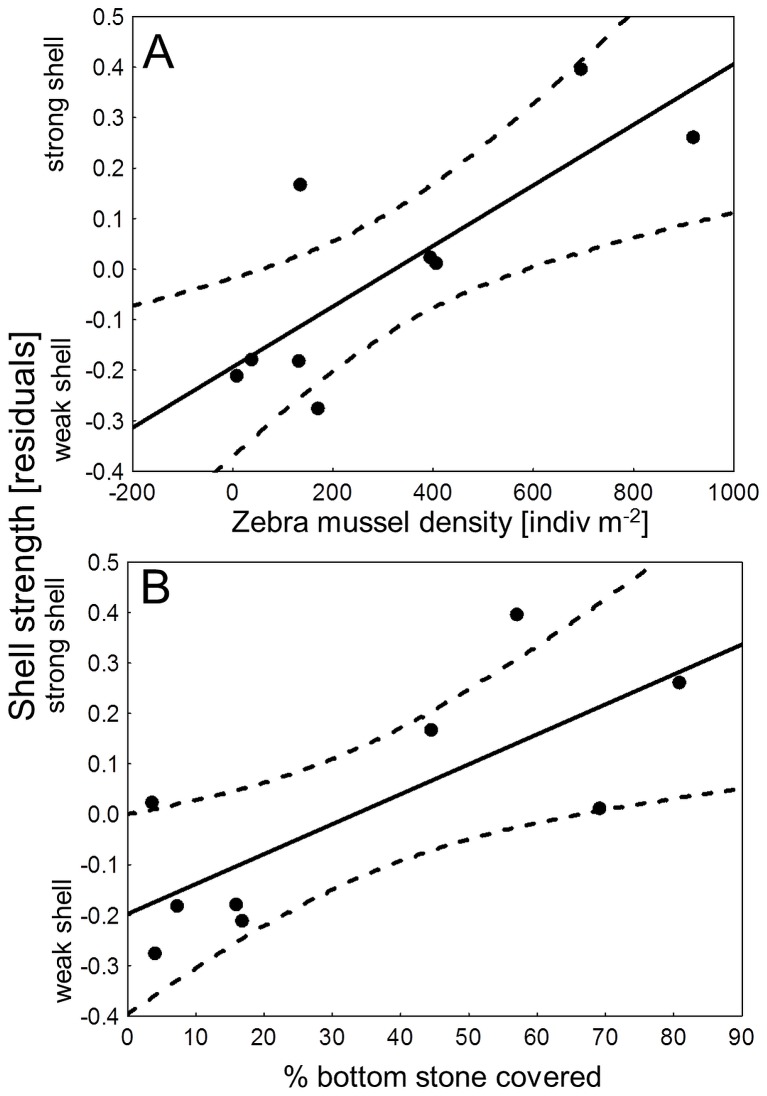
Population density and substrate influence shell strength in zebra mussels. Relationship between shell strength of zebra mussels and the two major shell strength determining factors (as identified by the PLS model): **A** intra-specific zebra mussel density and **B** percentage of bottom covered by stones. The r of the regressions of shell strength with the zebra mussel density are 0.8 (p<0.001) and with the percentage of lake bottom covered by stones 0.76 (p = 0.017).

The PLS model for shell strength had a R2Y of 0.83 and a Q2 of 0.58 and identified mussel population density (VIP value = 1.85) and percentage of bottom covered by stones (VIP value = 1.75) as the most important predictors explaining shell strength (S2 Figure). Shell strength increased with zebra mussel density (r = 0.80, p<0.001, Fig. 8) and with an increased percentage of stones as bottom substrate (r = 0.76, p = 0.017, Fig. 8). All other variables including crayfish population density showed lower VIP values (<1.0) and were hence of minor importance for explaining the zebra mussel shell strength (S2 Figure). The overall correlations among and between predictor and response variables are shown in loadings plots in the Supporting Information (S3 Figure for shape, and S4 Figure for strength). Separate models containing either PC1 or PC2 and a model comprising all three response variables did not perform better (all Q2 values were below 0.34). Mean age of mussels from the field was 4.6 years (±0.72 years SD) and no correlation between age and mussel shape (PC1, PC2) or any of the studied habitat or predator parameters was found (all r<0.55, all p>0.1).

## Discussion

In this study we found zebra mussels to respond equally to roach and crayfish predators with a non-specific anti-predator phenotype. This complies with our conceptual model, which predicts that if predators share a feeding mode *C*, the prey should express the response phenotype, *c*. We also found predator-specific responses against crayfish, which is in line with a specific expression of phenotypic responses *a* or *b* to opposing feeding modes from predator *A* or *B*. Phenotypes expressed in the combined and control treatment appeared to lie somewhere in the middle of those expressed in the crayfish and roach alone treatments. This connects to the conceptual prediction that a combination of predators A and B should lead to an intermediate phenotypic response *a-b*. Predator-specific phenotypes (shell shape and strength) expressed in the roach treatment did not significantly differ from the control or the combined treatment. The predator-specific responses were connected to trade-offs: faster growth resulted in a weaker shell. Thus, a stronger shell in response to a crushing predator also resulted in a reduced growth rate. Even though we saw evidence of the same trade-off in the field (growth vs. shell strength) we could not find any relationship between predator densities and mussel phenotype across several sites within a lake. Instead, intra-specific population density and bottom substrate appeared to affect phenotypes in the field.

### Trade-offs between anti-predator phenotypes and the effect of growth rate

Our results confirm the notion that there are trade-offs between defending against some predators and being susceptible to others [9], [27]. The adaptive value of phenotypic plasticity then depends on the prey's ability to express a phenotype that maximally protects against a combination of predators [25], [28]. Theory predicts that if multiple predators share a common feeding mode, the appropriate prey response is to increasingly express the defensive trait that provides protection against this common feeding mode [2], [29]. In support of this prediction we found that anti-predator responses that were non-specific and protected against both predators, did not differ between the single and combined treatments. In terms of our conceptual model, this means that the universal phenotypic response, *c*, and the intermediate response to predators, *a-b*, are largely identical. This similarity makes sense when considering the trade-offs: in *c* all energy re-allocated into defense is invested in optimal protection from both predators, whilst in *a-b* the prey equally optimizes its energetic investment by not allocating energy to defend against the wrong predator. Further tests of the dynamics of the prey's energetic budget will help determine whether the intermediate response is a response *per se*, or simply a by-product, i.e. predator-type unrelated growth.

Both predators used in our study have to overcome the mussels' attachment structures and remove the mussel from the substrate. Thus, in their initial step of prey-handling, crayfish and roach do not exhibit opposing feeding modes. Stronger attachment through more byssus threads decreases the threat of being detached from the substrate [30], [31]. Unlike responses in shell shape and strength, mussels did not show an integrated response in attachment when exposed to both predators simultaneously. Instead, mussels developed a similar strength of attachment in all predator treatments, which was significantly higher than in the predator-free control. Such non-specific defenses have been extensively studied and historically defined as on-off traits [9], [27], [29]. Our results showed that non-specific defenses were always ‘on’: there were a higher number of threads in all predator-treatments compared to the control. Non-specific traits can also come with trade-offs, because they also require tissue synthesis, but they would only exist in a binary, yes or no/on or off, response, and would not differ due to the presence or absence of particular predator types.

The situation is different for predator-specific defenses. We found a rounder and harder shell as a response to handling-time- and crushing force-limited predators. Whereas, the weakest and largest shells were observed in response to gape-size-limited predators, although this difference was not significant. Gape-size-limited fish are common predators for mussels and research on size-selective fish predation clearly demonstrates the reduced mortality of larger-sized mussels that are greater than the fish predator's gape-size [21], [32], [33], [34]. A round shell, on the other hand, protects against handling-time-limited predators as it appears to hamper handling and disperses crushing forces better across the whole shell [35], [36]. In addition to a round, hard-to-handle shape, the overall strength (the rate of calcification of the shell) provides protection against being crushed. Importantly, previous research found that a strong shell, rather than a large one, restricts consumption of mussel prey by handling-time-limited crushing predators [37], [38].

Trade-offs between growth and responses against predation risk are common in nature [12], [13], [39]. For example, zooplankton migrating deeper within the water column to escape fish predators trade increased survival from predation for decreased growth rate in the colder refuge waters [40]. In the same sense, zebra mussels grew faster to reduce vulnerability to the gape-size-limited roach. However, attainment of a size refuge from gape-size-limited predators inevitably comes with the cost of increased susceptibility to crushing predators such as crayfish. Recent studies suggest that developing a harder shell might simply be a passive by-product of mollusks reducing feeding, thus growth rate, in response to handling-time-limited predators [25], [41]. Indeed in an experiment similar to ours, Naddafi et al. [42] found that zebra mussels changed the clearance rate of some phytoplankton prey in response to cues from roach and crayfish. However, it remains unclear whether the relationship between growth rate and anti-predator traits is evidence for adaptive plasticity driven by predators.

This has bearings on the interpretation of the results from the control treatment. Although the prediction that byssus threads in the control treatments should be minimized relative to all other treatments was confirmed by our results, the implications of the results on shell strength and shape were unclear. We postulated that in the absence of predators the shell phenotype should represent one that allows for optimizing growth and reproduction. That said, the mussels continue to live in an environment that contains cues from conspecifics, as well as other relevant information influencing optimal energy allocation and phenotype expression. This complexity limits our ability to draw clear-cut conclusions about the nature of the phenotypes in the control treatment. Instead, the control phenotypes should be seen as a relevant benchmark against which other treatments can be compared. Future studies manipulating resources and predation risks from different predators will help elucidate what an optimal phenotype would look like, with or without energetic limitations. Such additional experiments are also needed to test for a growth rate vs. shell strength trade-off in the absence of predation risk and will help to reveal the mechanisms behind the expression of intermediate and no-predator responses.

### Assessment of predation threat influences anti-predator responses – the role of kairomones and alarm cues

The adaptive value of phenotypic plasticity depends on the prey's ability to accurately assess whether predators pose an opposing or unidirectional threat, as well as determining which phenotype to express in response to only one or a combination of predators [25], [28]. Recent research highlights that zebra mussels predominantly assess predation risk by sensing alarm cues emitted from crushed conspecifics [43]–[45]. Kairomones, cues emitted from predators even if these do not directly prey on mussels, have been found to play only a minor role as a trigger for anti-predator responses in zebra mussels [43]–[45]. This has ramifications for the interpretation of our results: we can postulate that, in combination, crayfish and roach should not produce any cues ‘alarming’ enough to induce more than non-specific defenses. Indeed, the combination of functionally inverse predators does lead to an expression of generalized non-specific defenses, as evidenced by the results of the attachment strength. The strong effect crayfish have on defense phenotypes in mussels might result from the presence of alarm cues released by the crayfish-typical feeding mode. Crayfish, which crush mussels externally, likely release more alarm cues than roach, which swallow whole mussels and then crush them internally [44], [46]. This difference in alarm cues released by mussel-crushing vs. mussel-swallowing predators has frequently been found to influence defenses in bivalve prey [44]. Experimental tests confirmed that even artificially crushed mussels release alarm cues that trigger defenses [44], [46]. The defenses in response to artificially crushed mussels were weaker or even absent if mussels perceived merely the presence of predators (i.e. kairomones without alarm cues) [43], [46]–[48]. In the control, roach only, and combined treatments there might not have been enough mussels crushed externally to trigger a specific defense. In the roach treatment none of the prey-mussels fed to the predator were crushed externally. In the control treatment no mussel was crushed at all. The trend towards a more elongated and weaker shell in the roach treatment could be explained either by the presence of kairomones that were absent in the control or the effect of mussels crushed internally, both resulting in enough cues for the mussels to develop a mean phenotype marginally different from the control or combined treatment. In the combined treatment, roach fed on some mussels leaving fewer mussels for crayfish to crush externally. This might explain why the combined treatment triggered no predator-specific response to crayfish. The presumably stronger response of mussels to alarm cues rather than to kairomones is also relevant for our field study. The importance of alarm cues supports our claim that assessing the predation risk as density of predators that are large enough to consume mussels is reasonable. Only predators that are large enough to crush mussels are likely to trigger defenses that are predator-specific [45].

### Prey phenotypes in the wild depend on more than anti-predator responses

In our study we found evidence for trade-offs in the wild corresponding to those seen in the experiment (growth rate vs. shell strength). But we found no evidence for the influence of predation risk on the phenotypic variation observed in the field. Instead, we found intra-specific density and the bottom substrate to influence prey phenotypes. Intra-specific density is generally tightly connected to the strength of competition [49]. For sessile organisms like mussels, a high intra-specific density can lead to competition for space or nutrients. This might decrease growth rate, especially on stones where mussels are typically tightly packed [50]. Our results from the field further support the idea that growth rate might function as an important mediator of morphological defenses [25]. This also strengthens the results from our lab experiment, in which we could link phenotypes to growth rate directly by measuring mussel size pre-and post-treatment. However, measuring attachment strength in the field proved to be impossible. Mussels in natural communities are frequently clumped together and their byssus threads then build web-like structures that make them hard to count. Entanglement of threads also leads to attachment strengths that are much higher than the simple relationship between number and strength suggests for a single mussel (own unpublished data). Recent empirical work on phenotypic plasticity highlights the influence and interaction of abiotic factors with predator and prey density to shape prey phenotypes [18]. For example, the degree and shape of plastic phenotypic responses in tadpoles was found to be contingent on hatching pond desiccation as an important abiotic factor [51], [52]. By combining a wide range of relevant biotic and abiotic factors our study further exemplifies that factors found important for phenotype expression under controlled conditions may not suffice to explain phenotypic variation found in the wild.

### Conclusions

Our results show that two different predators can exert both functionally equivalent and inverse selection pressures on a single prey. The prey seems to be able to respond accordingly by expressing a non-specific trait in all situations of predation risk, specific traits when facing a single predator alone, and an intermediate trait when facing combined predators, a predator emitting less alarm cues, or no predator at all. Our finding of intermediate phenotypes in response to a combination of predators, but also in the absence of any predator at all, contributes to the growing evidence that the more natural situation of multiple predators has complex influences on prey [3], [53]. However, because in the wild any prey and predator is part of a complex community, phenotypic variation found in field surveys can easily contradict predictions from simplified experimental conditions.

## Materials and Methods

### Study site

The study was conducted at the field station at Lake Erken in southeastern Sweden (59°51′N, 18°36' E), with approval by Uppsala University's ethics committee (approval ID C231/10) . The study did not involve endangered or protected species. Lake Erken's field station is part of SITES (Swedish Infrastructure for Ecosystem Science), which should be contacted for future permission. The lake's trophic status is meso-eutrophic (Total Phosphorus: 24–51 µg L^−1^), it has a surface area of 24 km^2^ and a mean depth of 9 m. Lake Erken has no major in or outflow, its pH is well buffered due to the local geology and the lake water has a Ca^2+^ concentration of approx. 40 mg L^−1^ [54]. Consequently, we assume the long term mean of chemical habitat parameters, such as carbonate concentration, to be equal between sites. Zebra mussels were first recorded in the lake in 1975 and signal crayfish were deliberately introduced between 1966 and 1969. Both species established a lake-wide population within years after introduction and together dominate the benthic community in terms of biomass [22], [42]. Roach is native to the lake and the second most abundant fish species in terms of biomass (30% of total fish biomass). The most abundant fish is perch (60% of total fish biomass), which does not feed on zebra mussels [55]. Other cyprinids occurring in the lake that could potentially be mussel predators are the common and white bream (*Abramis brama* and *Blicca bjoerkna*) and tench (*Tinca tinca*). Previous studies suggest that local roach populations control zebra mussel population density via predation [56]. Stable isotope data suggests that zebra mussels constitute up to one third of the diet of the lake's signal crayfish (Hirsch and Svanbäck, unpublished data). Importantly, long-term fisheries data from the lake suggests that there are parts of the lake that contain consistently more dense populations of either roach or crayfish, or both ([56], unpublished data from the research station at Lake Erken and personal communication from fishermen). Hence, areas with a high or low density of either predator are likely to represent consistent localized differences in predator pressure. More mobile predators like diving ducks of the genus *Aythya*, *Bucephala* and *Haematopus* spp., which are important predators of zebra mussels in other lakes, occurred in Lake Erken in densities of less than one breeding pair per lake surface km^2^ over the past 30 years [57]. A profound influence of wintering bird predation on mussels is precluded by the lake's ice-cover, which typically lasts from November through April. Consequently, we assume that roach and signal crayfish are the most important predators of zebra mussels in the lake and that predation pressure from these two predators differs locally within the lake.

### Experiment

For the experiments we sampled around 700 same-sized mussels by carefully removing them from stones in 0.5 to 1.5 m water depth in a littoral area of approximately 20 m^2^. We acknowledge that many studies on phenotypic plasticity use clones or laboratory strains of study species. However, recent research on the expression of phenotypic plasticity highlights that individuals from wild-populations express traits under ecologically relevant conditions in the lab and that wild-caught individuals might be more likely to exhibit natural anti-predator responses than laboratory strains [58], [59]. Prior to the start of the experiments the mussels were kept in a cylindrical cage in the lake near the sampling site at 1 m depth for a maximum of 24 h. We caught roach by fishing with non-barbed hooks, and crayfish by baited crayfish traps from the same site as the mussels came from. All predators were kept for a maximum of three days in carp keep nets (roach) or a cylindrical cage (crayfish) prior to the experiments and fed (*ad libitum*) with mussels having the same size as the experimental mussels.

We conducted the experiments in 28 (4 treatments x 7 replicates per treatment) 96 L overflow tanks. The four predation treatments were *i*) Roach only (one roach per tank, mean weight 59.86±24 g, mean ± SD) *ii*) Crayfish only (one crayfish per tank, mean weight 33.65±3.5 g), *iii*) Combined predator treatment (one roach plus one crayfish per tank, roach weight: 66.7±22.6 g, crayfish weight: 39.52±8.35 g), and *iv*) Control treatment: no predators present in the tanks. The predator densities applied are thought to reflect natural densities in the field [42]. It should be noted that the biomass of roach and crayfish predators did not differ between single and combined treatments (T-test, df  = 7, roach: T = −1.71, p>0.1, crayfish: T = −0.55, p>0.1) whereas the total predator biomass was significantly higher in the combined treatments than in the single predator treatments (T-test, df  = 7, roach: T = −3.18 p<0.01, crayfish: T = −5.62, p<0.01). We believe this reflects the natural situation in the wild, where predators do not substitute each other.

All experimental tanks were supplied by lake water coming from 4 m depth. A tank water renewal rate of 4 h 40 minutes ensured well-oxygenized conditions for all animals and the inflow rate of 5.7 mL s^−1^ (well exceeding the average filtration rate of zebra mussels of 0.01 mL s^−1^ [60]) precluded any form of food deprivation or competition among the mussels. Temperature was kept constant at 18±1°C by means of thermostats. This temperature reflects littoral water temperatures during the growing season and hence the natural growth temperature of mussels in the lake. In all tanks (including control tanks) we added an 8×15cm PVC pipe to which we fixed floating PVC threads. Roach used the threads as a macrophyte-like cover and crayfish hid in the pipe.

To start the experiment 16 mussels were randomly picked from the cage, their byssus threads carefully removed (to ensure *de novo* production of threads uninfluenced by pre-experimental conditions), their size measured (length from the anterior to the posterior margin, width inflation at the broadest part of the shell, height from the ventral side to the dorsal margin) to the nearest 0.01 mm using a digital vernier caliper, then they were placed next to one another into the middle of a 30 cm^2^ surface area consisting of four unglazed chime tiles. Tiles were placed into the tanks two days prior to the experiment to allow biofilm formation. After the mussels were placed onto the tiles they were covered with a wire cage (32.5×32.5×10 cm, mesh size 3 mm) to protect them from predators. All predators were weighed and then put into the tanks immediately afterwards, according to the treatments. This set-up closely matched the situation of both predators feeding over a mussel bank in the lake and prioritized the transferability of our set-up to the field over the ability of predator diet control [61], [62].

At the start of the experiment the mussels had a length of 11.5±0.9 mm, width of 5.9±0.45 mm, and a height of 5.88±0.53 mm (mean ± SD). Mussel size at the start of the experiment did not differ among tanks (all pairwise comparisons insignificant) except for one control tank in which mussels were slightly larger than in the rest of the tanks (pairwise comparisons with other tanks p<0.05). We deemed our analyses robust against this single outlier because that one tank constituted only 1.3% of all data. Hence, this data was kept to maintain a balanced design. Mussel growth was determined by measuring the size in the three dimensions (length, width, and height) before and after the experiment. Every third day we fed predators with four small mussels per tank (including the combined treatments), similar in size to the experimental mussels, and cleaned the tank of prey mussel remains and predator feces on the following day. Because the predators leave characteristic remains when consuming zebra mussels [21], [22], visual inspection of the remains allowed us to confirm that all predators consumed prey in all treatments. Naturally, the water from the lake might also contain “background cues”. However, we expect these cues to not significantly affect our results because all tanks received the same water and we wanted to simulate natural conditions in the tanks. Recent research argues that the completely predator-cue-free maintenance conditions frequently used in laboratory experiments, can limit the researchers' ability to detect relevant plastic anti-predator responses [63]. After five weeks we terminated the experiment and assessed the strength of attachment to the surface. We assessed attachment strength indirectly by counting the number of byssus threads radiating from the central stalk of the byssus glands to the nearest five threads under a dissection microscope (45x magnification) after carefully removing the mussels from the substrate with a scalpel. Following Leonard et al. [47], we used the number of byssus threads as a proxy for attachment strength. All mussels were subsequently frozen at −20°C and stored until further analysis.

### Field survey

Mussels and predators were sampled at nine sites equally distributed across the lake (S5 Figure). At these sites we used four multi-mesh Nordic gill-nets to sample roach, and 20 crayfish traps (see [64] for trap details) baited with dead cyprinid fish to sample crayfish. All field sampling took place in the beginning of July which ensured that fish and crayfish were active and not in spawning or molting season. One set of nets (1.5 m deep and 30 m long) and ten traps were set at 2 m depth just outside of the vegetation in the littoral zone and another set of two nets (6 m deep and 27.5 m long) and 10 traps were set in approx. 200 m distance from the shoreline towards the deepest part of the lake. All nets and traps were set in the afternoon and removed 16–18 hours later, the following morning. Fish were measured and weighed immediately after catch and crayfish were immediately frozen at −20°C for later analysis.

Roach need to develop a mouth large enough for ingesting mussels that can then be processed by the pharyngeal teeth. Roach >150 mm TL have been found to readily feed on zebra mussels [65]. Thus, we expressed roach predator threat as catch (biomass, g) per unit effort (CPUE) of roach larger than 150 mm. We also included the CPUE (biomass) of other potentially zebra mussel consuming fish (bream and tench >150 mm) as a separate factor of predator threat. Crayfish traps usually catch crayfish >60 mm total length. We found crayfish of this length to consume zebra mussels in the lab and hence used the biomass (g) of all crayfish caught as the catch per unit effort (CPUE). The size-thresholds we chose for quantifying predation risk are based on the fact that alarm cues are the most important trigger for the expression of anti-predator defenses in zebra mussels [43]. If predators crush prey over a mussel bed the other mussels can sense the alarm cues from their conspecifics. This then triggers their anti-predator responses. In addition, recent research has shown that zebra mussels can modulate their plastic responses to different sizes of predators: mussels responded less to small roach and perch than to large roach capable of crushing mussels [45].

Naturally, there are factors other than predation that might influence mussel phenotypes in the field. In addition to predator threat we assessed intra-specific density, bottom structure, wave exposure and resource density as factors presumably relevant for phenotypic variation in mussels. For sessile organisms like mussels, intra-specific density can entail competition for space, which impairs growth rate and might affect phenotypic variation [50]. We sampled zebra mussels by retrieving five Ekman grabs from approximately 2 m water depth (the predominant habitat of zebra mussels in the lake [56]) at each site. Where rocks made successful Ekman grabs impossible we handpicked mussels by snorkeling, or used a kick sampler to collect mussels from a defined area of the bottom. Mussels were detached from the substrate directly after catch and then frozen and stored at −20°C for later analysis. We also estimated intra-specific density in zebra mussels with video transects. For each transect we drove an engine-powered boat, parallel to the shoreline and close to the exposed traps and nets, at a constant speed of 2 km h^−1^ for 200–250 m. Using a hand-held camera pointing into a bathyscope, we recorded the bottom structure including the zebra mussels in a defined area (2 m^2^) at 2 meters depth. Using the free software ImageJ (available at http://rsb.info.nih.gov/ij; developed by Wayne Rasband, National Institutes of Health, USA) we froze the film frames after every 2 m^2^ were renewed, counted the visible zebra mussels and analyzed the bottom substrates for each consecutive frame. Density of zebra mussels assessed by the video transects correlated significantly with the density estimate from the Ekman-grabs (r = 0.85, p<0.01), and was congruent with a previous density study using SCUBA diving transects at fewer sites [56]. Thus, we used this data in all analyses as it incorporated a larger area than the Ekman-grabs.

Phenotypic variation in mussels has been suggested to depend on the bottom substrate, with different substrates varying in suitability for juvenile or adult mussels [66], [67]. We assessed the bottom substrate and classified it as percentage of the area of the 2 m^2^ frame from the video transect recordings covered with stones (including pebbles and rocks), plants (all submerged macrophytes) or soft bottom (sand, silt, detritus). Phenotypic variation of mussels in marine systems is influenced by wave action [66], [68]. Water currents created by wave action can exert forces on the shell that inhibit growth in height and can lead to rounder shells [69]. Therefore, we calculated the different sites' wave-exposure as the effective wind fetch of each site in kilometers (a common surrogate parameter for wave exposure [70]) using a detailed lake map following the standard procedure developed by Howes, et al. [71].

The major food source for zebra mussels is phytoplankton [72]. Changes in the availability of this resource could influence the mussels' growth rate and this in turn could affect phenotypic variation among sites. Therefore, as a proxy for phytoplankton density we measured the chlorophyll-a (Chl-a) concentration (µg L^−1^) at all sites on three occasions with 10 day intervals in the growing season (starting in July). We sampled water in 1 m intervals from 1 to 8 meters depth, both near the littoral and the more offshore exposed nets and traps, integrating over the whole water column. Water samples were filtered onto a glass fiber filter (MGC, pore size  = 1 µm) directly after sampling and frozen at −80°C until analysis, which followed the protocol ISO 10260 [73] for spectrometric determination of chlorophyll-a concentration. In the Supporting Information we provide all data on environmental factors and mussel traits collected in the field (S1 Table, S2 Table).

### Morphological analyses and size measures

We assessed the shell shape of mussels by means of Fourier shape analysis implemented in the free statistical software SHAPE v.1.3 (available at http://lbm.ab.a.u-tokyo.ac.jp, developed by Hiroyoshi Iwata, University of Tokyo, Japan). This method captures the shell shape as a contour based on 20 elliptic Fourier descriptors and generates principal components that can be visualized and explain an object's size-independent shape [74]. As input for the program we used digital picture files from all mussels (from the experiment and from the field) that were created by placing the right valve of each individual mussel on a flat-bed scanner. Based on this input the software generated 6 principal components (PCs) that explained the total variation in shell shape across all individuals (S6 Figure). After visualization of these components we chose the first two PCs as representing biologically meaningful phenotypic variation in shells. Both PC1 and PC2 accounted for 72% of the total variation (PC1  = 63%, PC2  = 9%) and were clearly interpreted as depicting a round or elongated shell shape (S6 Figure). The remaining principal components each explained less than 6% of the variation and were excluded from the analyses (S6 Figure).

We aimed at using a size measure that would be unbiased towards only local extensions of the mussel shell. Therefore, we did not simply use shell length as is commonly done. Instead we expressed mussel size as a principal component (explaining 97% of the total variation, eigenvalue 2.3) derived from all three size dimensions (length, width, height). Because elliptic Fourier descriptors do not contain size information there is no need to standardize the outlines to a standard size [67]. Yet, our shell shape measures PC1 and PC2 did show a weak relationship with shell size (experiment and field mussels: all r<0.19). We therefore accounted for the size-shape relationship in our analyses by using size as a covariate or by using individuals' residual values from the size-phenotype correlation (see below).

### Shell strength analyses

We used a force gauge (Mark 10 motorized test stand ESM 3000) to measure the crushing resistance and hence the shell strength of all mussels after scanning them. Prior to the crushing we placed the mussel shells onto the test stand so that the ventral margin (the area of the valve where signal crayfish crush zebra mussel shells [22]) pointed perpendicular to a blunt needle that was fixed to the force sensor (SSM10, accuracy ±0.15 N) of the gauge. The force gauge then moved the needle onto the shell at a constant speed of 0.25 m minute^−1^ until the shell broke. We used the mean of the resistance to breakage from the same shell area at both valves as a measure of shell strength. We acknowledge that this is not the strongest part of the shell but it is the part where crushing predators breach the shell, thus we accepted the strength at this point to represent a relevant measure of the shell strength as an anti-predator defense. In both the field and the experiment we found shell strength to be strongly positively correlated to mussel size (correlation coefficients for field and experimental data both>0.75, p<0.001).

### Growth measurements

For the experimental mussels we calculated growth as size increase of a shell in all three measured shell dimensions by measuring all mussels before and after the experiment (see above). For mussels in the field we back-calculated growth, in the first year post-larvae settlement, from growth increments using a method similar to determining fish growth from opercular bones [75]. Inferring age and growth by winter band growth increments of the shell is a long-established method in bivalves [69], [76] and has successfully been used for determining the age and growth of zebra mussels [77], [78]. We used the digitally scanned pictures and measured growth increment lengths with ImageJ. Instead of counting the increments on the outside of the shell, we quantified the winter bands on the inside of the shell as we deemed them to be more clearly distinguishable. For growth rate back calculation we used a conservative 597 individuals (a few individuals' growth increments were not unambiguously identifiable because of abnormalities in the pigmentation of the shell). Both shell shape and shell strength analysis were conducted with the total of 1202 mussels (446 individuals from the experiment and 84 individuals for each of the nine sampling sites). Since we could only obtain 84 individuals at one site we randomly selected 84 individuals from the other sites where we sampled more mussels.

### Statistical analyses

To test for differences in the experiment we conducted nested ANCOVAs using the ‘variance components' function in Statistica (Vers. 12) with a hierarchically nested model. Nesting codes identified overall consecutive levels such that tanks with individual mussels were a random factor and treatments the categorical predictors. We also included shell size as a covariate. We used individual mussels' values for PC1, PC2, shell strength, and number of byssus threads from each tank as response variables. For growth we did not use size as a covariate as we deemed these traits to be a direct function of size (growth). Since we could not obtain data on an individual level for growth we used one mean value per tank and conducted a regular ANOVA with the treatments as predictor variables. All significant omnibus tests were followed by Fisher's LSD Post-hoc tests. To assess which factors determine the phenotype (PC1, PC2 and shell strength) of zebra mussels in the field, we built two PLS (Partial least squares) models. PLS models do not allow correction for a single covariate as in ANCOVAs. Therefore, to account for possible size-dependence of shell strength and shape in the PLS models on the field data we used residual values. The residual values were obtained from the regression of each individual's shell strength and shape (PC1 and PC2) with size. Thus, we could generate a size-independent measure of the mussels' phenotypes at each site by calculating the mean of the residuals for each site. The first PLS model we constructed comprised both PC1 and PC2 and the second one had shell strength as response variable. The goodness of fit of a PLS model is expressed in the terms RY and Q2. RY denotes the percent of the variation of all the response variables that are explained by the model and Q2 is a measure of the predictive power of the model. There are no strictly defined thresholds for when to accept Q2 values in PLS model validation as significant or insignificant. To identify predictor variables with importance for the model and hence highest influence on the response variables we calculated the VIP (variable influence on projection). In a VIP analysis all variables with a numeric value larger than 1.0 have a significant influence on the response variables [79]. To better visualize the direction of influence on the response variables we provide linear regression plots for the most important predictor variables. Predictor variables included in the models were CPUE of roach, other fish predators and crayfish predators, zebra mussel density, bottom substrate (see description in the *Field survey* paragraph), Chl-a concentration, and wave exposure. For the PLS modeling we used SIMCA 12.0 software (Umetrics AB).

## Supporting Information

S1 Figure **VIP from the PLS model for PC1 and PC2.** Influence of the environmental predictor variables on the shell shape as a response variable (PC1 and PC2 describing mussel shell shape). Influence visualized in the form of variable influence on projection (VIP) of the first component in the PLS model including PC1 and PC2.
(TIF)

S2 Figure **VIP from the PLS model for shell strength.** Influence of the environmental predictor variables on the shell strength as a response variable (shell strength as the mean for each site of the residuals from the correlation between shell size and strength across the whole data set). Influence visualized in the form of variable influence on projection (VIP) of the first component in the PLS model.
(TIF)

S3 Figure **Loadings scatterplot PLS model for PC1 and PC2.** The scatterplot depicts the factor loadings for the PLS model including the two principal components explaining mussel shell shape (PC1 and PC2). The plot visualizes the calculated weights (**w**, **c**) for the first two components w*c[1) and w*c[2] explaining the relation between the shape (PC1 and PC2 as response variable depicted as squares) and the potential predictor variables included in the model (predictor variables depicted as triangles). The weights for the predictor variables (**w**) describe the contribution of each predictor variable to the relation between predictor variable and response variable and congruently the weights for the Response variables (**c**) describe the influence of response variables on predictor variables. For interpretation of the plot one draws a line from a response variable through the origin (where zero lines meet) and then projects the predictor variable of interest onto that line. predictor variables furthest away from the origin have the largest influence on the response variable. Predictor and response variables located in proximity to each other are positively correlated with while variables located at opposite sides of the origin are negatively correlated.
(TIF)

S4 Figure **Loadings scatterplot PLS model for shell strength.** Loadings scatterplot for the PLS model including the shell strength in the form of the residuals from the correlation between shell size and strength. The plot visualizes the calculated weights (**w**, **c**) for the first two components w*c[1] and w*c[2] explaining the relation between the shell strength and the potential predictor variable included in the model (predictor variables depicted as triangles). The weights for the predictor variables (**w**) describe the contribution of each predictor variable to the relation between predictor variable and response variable and congruently the weights for the Response variables (**c**) describe the influence of response variables on predictor variables. For interpretation of the plot one draws a line from a response variable through the origin (where zero lines meet) and then projects the predictor variable of interest onto that line. Predictor variables furthest away from the origin have the largest influence on the response variable. Predictor and response variables located in proximity to each other are positively correlated with while variables located at opposite sides of the origin are negatively correlated. As can be inferred from the plot the predictor variables “mussel density” and “% bottom stone covered” have the biggest influence on mussel shell strength.
(TIF)

S5 Figure **Map of the study lake with the nine sampling sites.**
(TIF)

S6 Figure **Visualization of contour shapes.** Contour shapes are visualized as the 6 effective components from the elliptic Fourrier analysis in SHAPE v 1.3 explaining 99.8% of the variation in the total data.
(TIF)

S1 Table **Raw data for PLS model.** Data on factors tested in the PLS to explain zebra mussel phenotypes in the field.
(PDF)

S2 Table **Raw data for mussel field data.** Data on shell shape and strength and size of zebra mussels in the field.
(PDF)
