# Supplementary figures and images for: Plastic Responses of a Sessile Prey to Multiple Predators: A Field and Experimental Study
Source: PLoS One. 2014 Dec 17;9(12):e115192. doi: 10.1371/journal.pone.0115192 (PMC4269437; doi:10.1371/journal.pone.0115192)

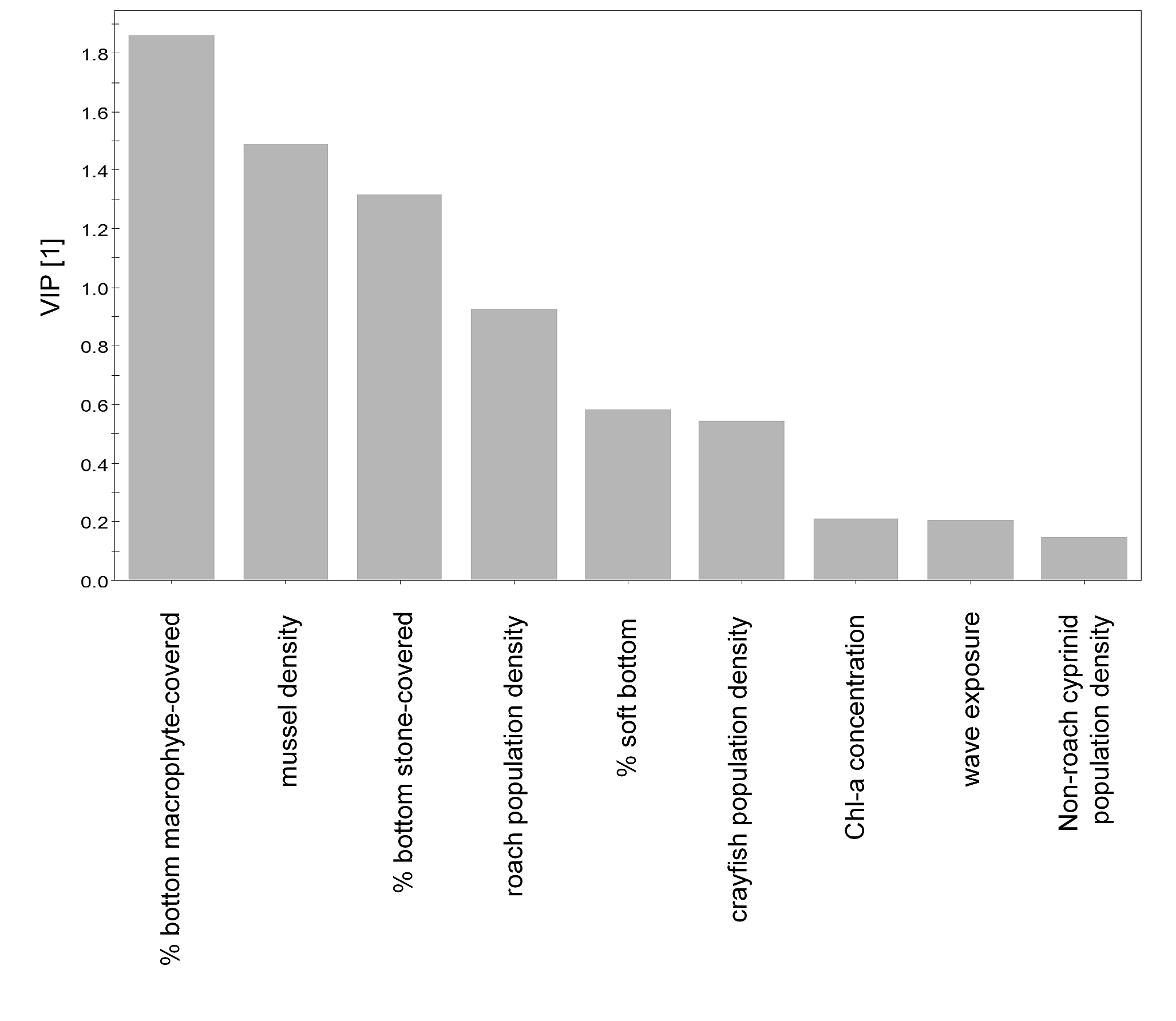

Supplement: S1 Figure — VIP from the PLS model for PC1 and PC2. Influence of the environmental predictor variables on the shell shape as a response variable (PC1 and PC2 describing mussel shell shape). Influence visualized in the form of variable influence on projection (VIP) of the first component in the PLS model including PC1 and PC2. (TIF) [file pone.0115192.s001.tif]

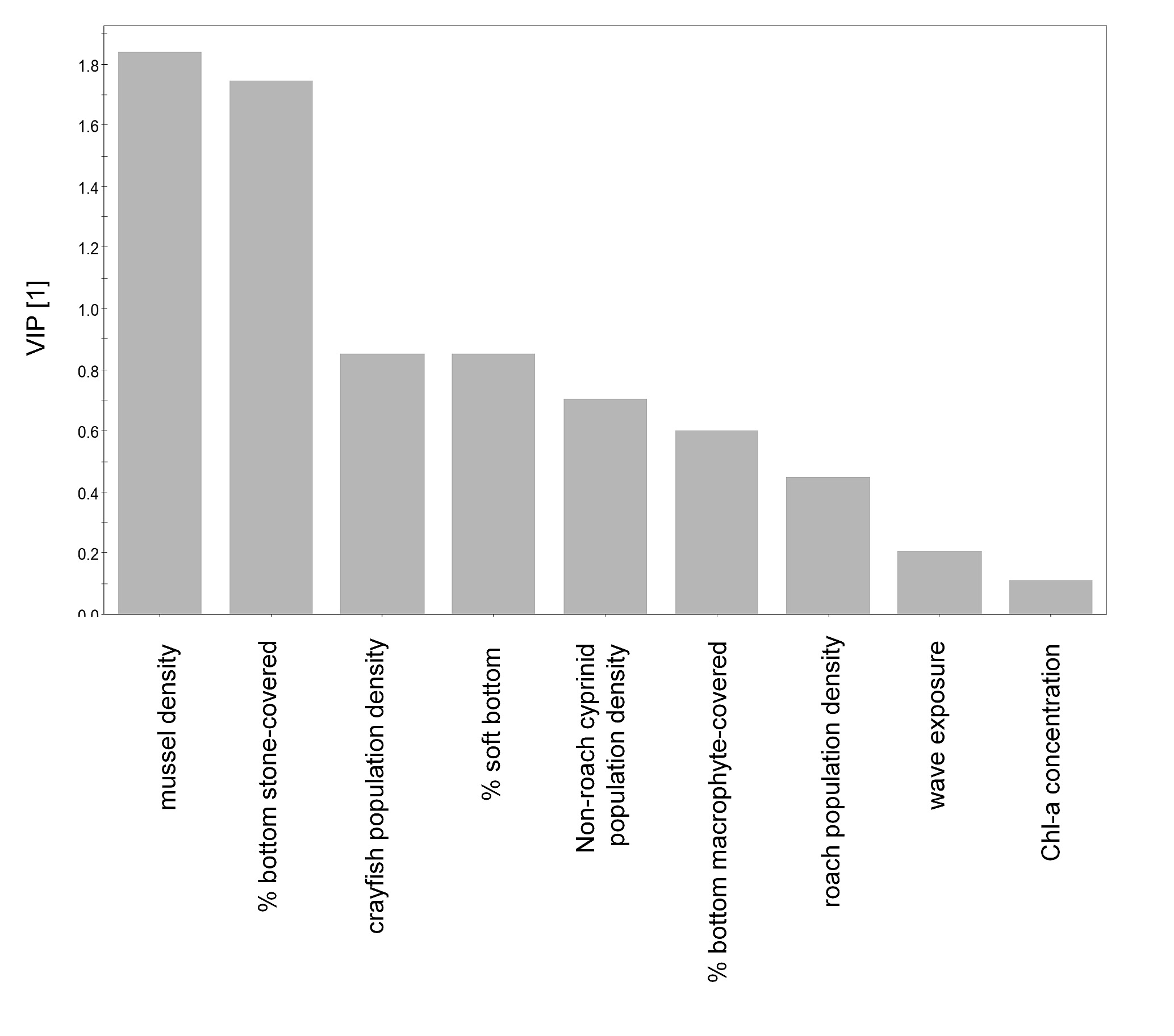

Supplement: S2 Figure — VIP from the PLS model for shell strength. Influence of the environmental predictor variables on the shell strength as a response variable (shell strength as the mean for each site of the residuals from the correlation between shell size and strength across the whole data set). Influence visualized in the form of variable influence on projection (VIP) of the first component in the PLS model. (TIF) [file pone.0115192.s002.tif]

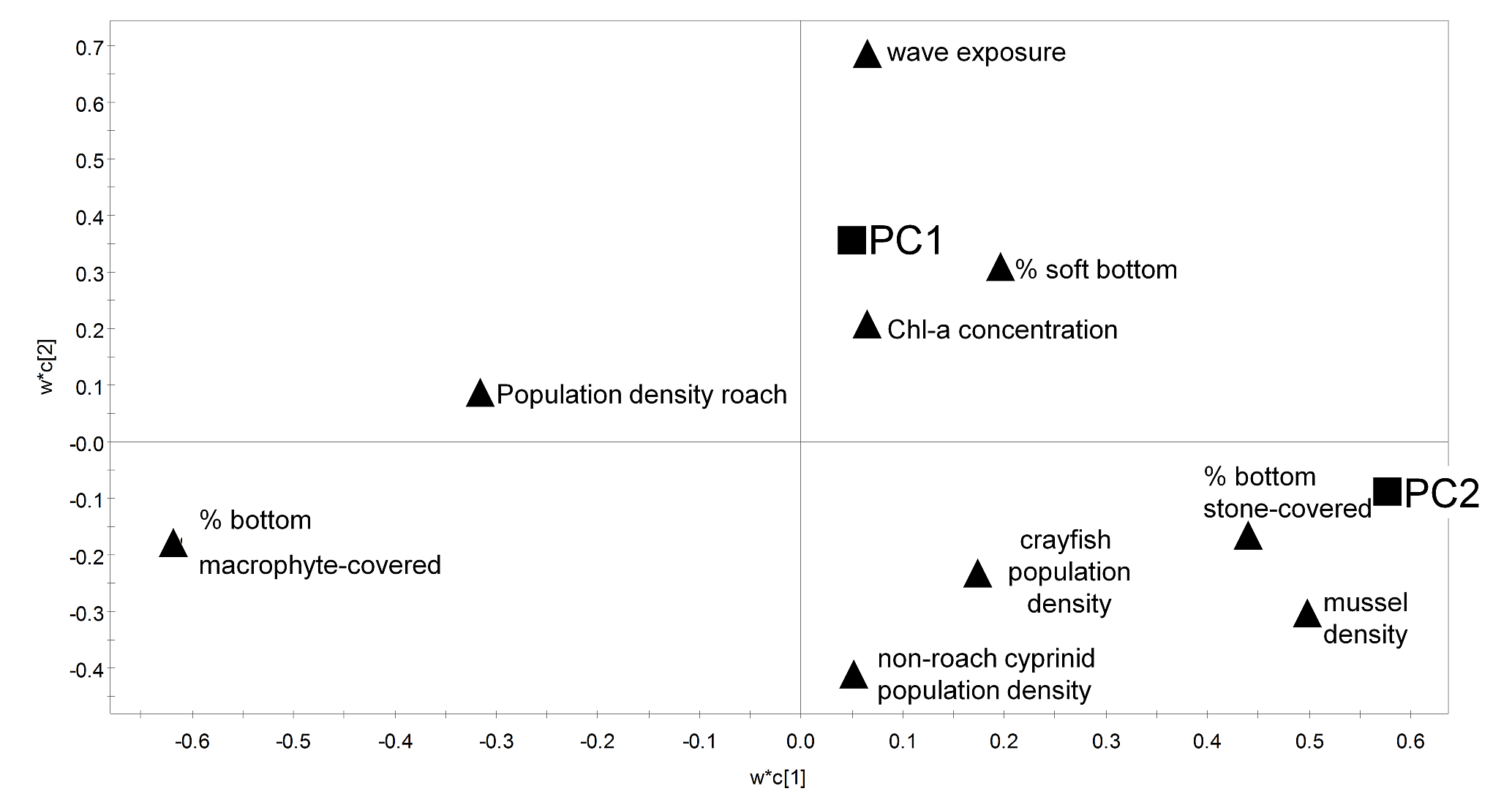

Supplement: S3 Figure — Loadings scatterplot PLS model for PC1 and PC2. The scatterplot depicts the factor loadings for the PLS model including the two principal components explaining mussel shell shape (PC1 and PC2). The plot visualizes the calculated weights (w, c) for the first two components w*c[1) and w*c[2] explaining the relation between the shape (PC1 and PC2 as response variable depicted as squares) and the potential predictor variables included in the model (predictor variables depicted as triangles). The weights for the predictor variables (w) describe the contribution of each predictor variable to the relation between predictor variable and response variable and congruently the weights for the Response variables (c) describe the influence of response variables on predictor variables. For interpretation of the plot one draws a line from a response variable through the origin (where zero lines meet) and then projects the predictor variable of interest onto that line. predictor variables furthest away from the origin have the largest influence on the response variable. Predictor and response variables located in proximity to each other are positively correlated with while variables located at opposite sides of the origin are negatively correlated. (TIF) [file pone.0115192.s003.tif]

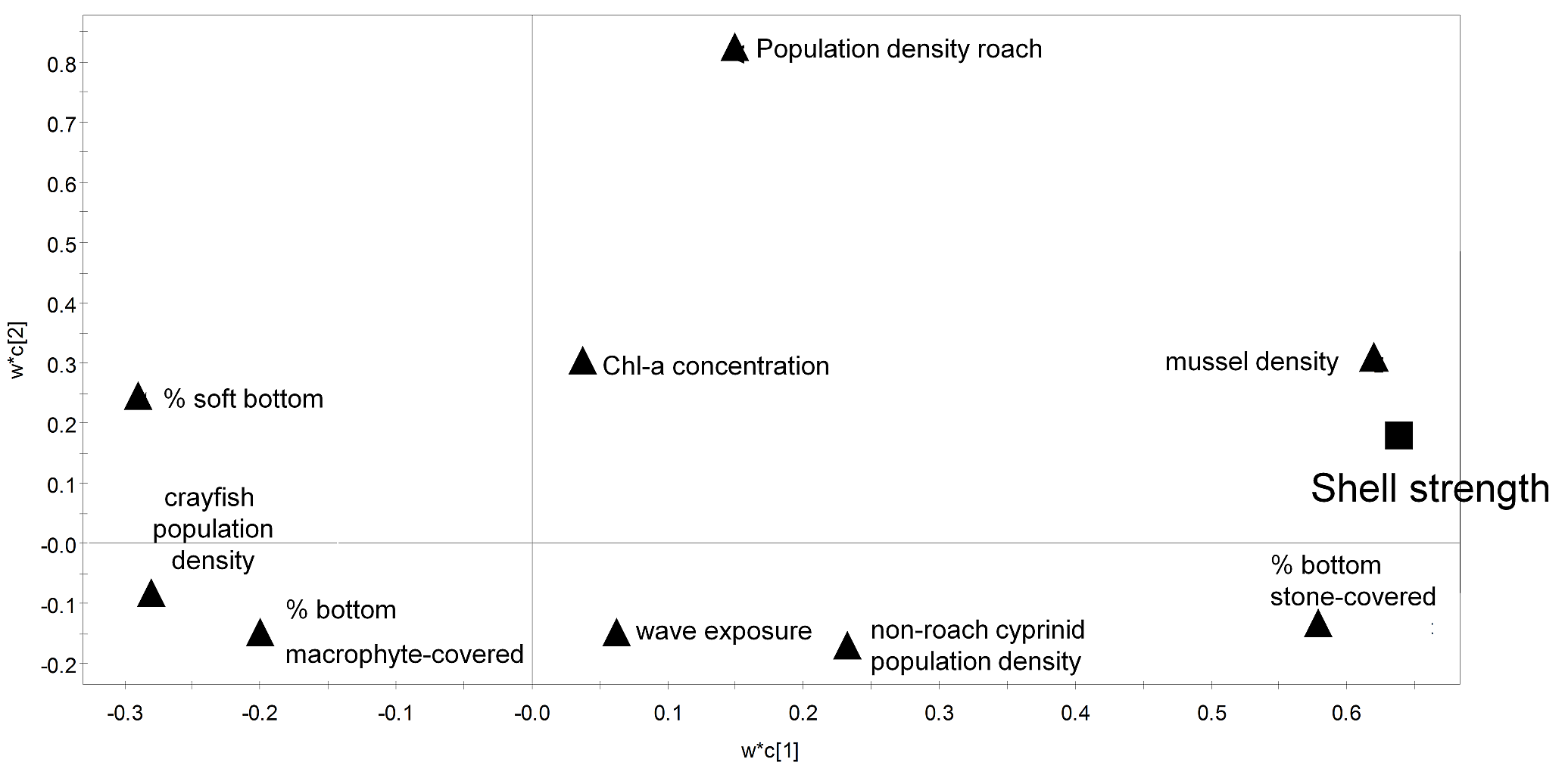

Supplement: S4 Figure — Loadings scatterplot PLS model for shell strength. Loadings scatterplot for the PLS model including the shell strength in the form of the residuals from the correlation between shell size and strength. The plot visualizes the calculated weights (w, c) for the first two components w*c[1] and w*c[2] explaining the relation between the shell strength and the potential predictor variable included in the model (predictor variables depicted as triangles). The weights for the predictor variables (w) describe the contribution of each predictor variable to the relation between predictor variable and response variable and congruently the weights for the Response variables (c) describe the influence of response variables on predictor variables. For interpretation of the plot one draws a line from a response variable through the origin (where zero lines meet) and then projects the predictor variable of interest onto that line. Predictor variables furthest away from the origin have the largest influence on the response variable. Predictor and response variables located in proximity to each other are positively correlated with while variables located at opposite sides of the origin are negatively correlated. As can be inferred from the plot the predictor variables “mussel density” and “% bottom stone covered” have the biggest influence on mussel shell strength. (TIF) [file pone.0115192.s004.tif]

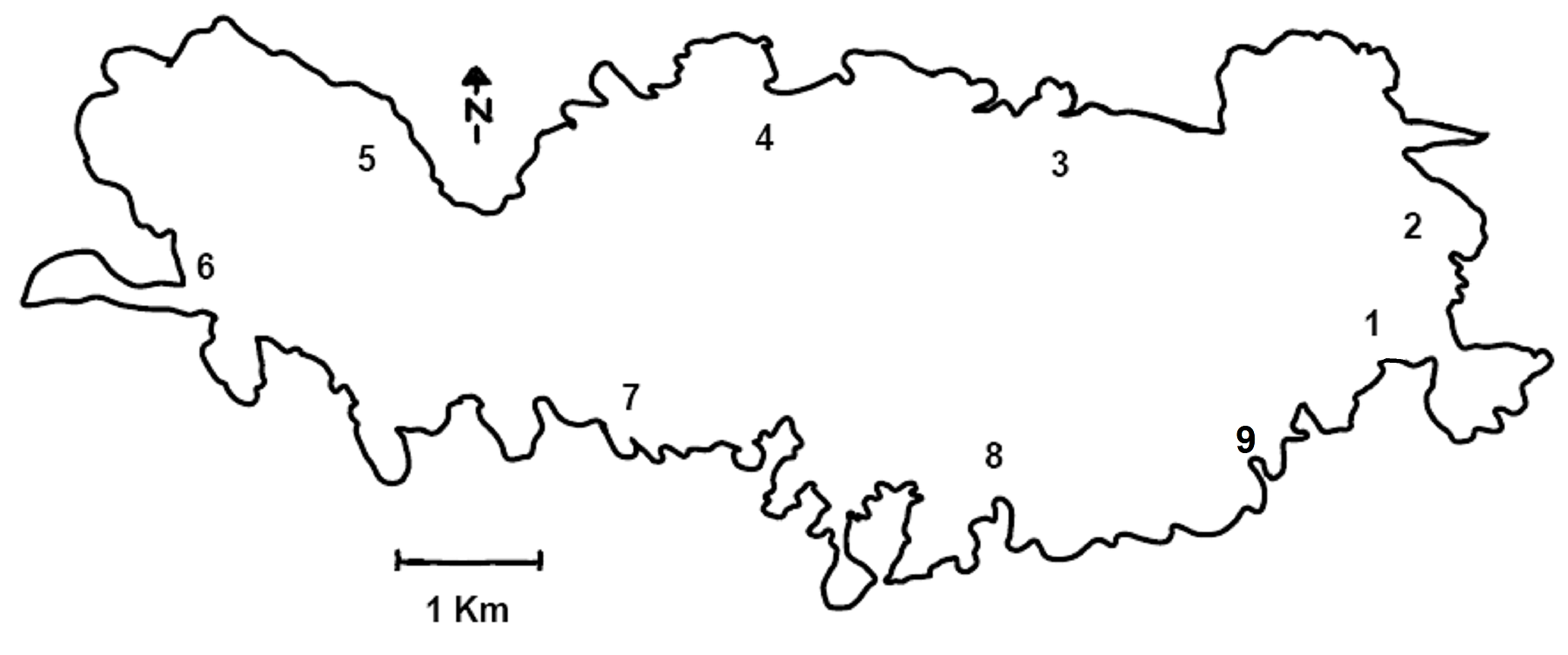

Supplement: S5 Figure — Map of the study lake with the nine sampling sites. (TIF) [file pone.0115192.s005.tif]

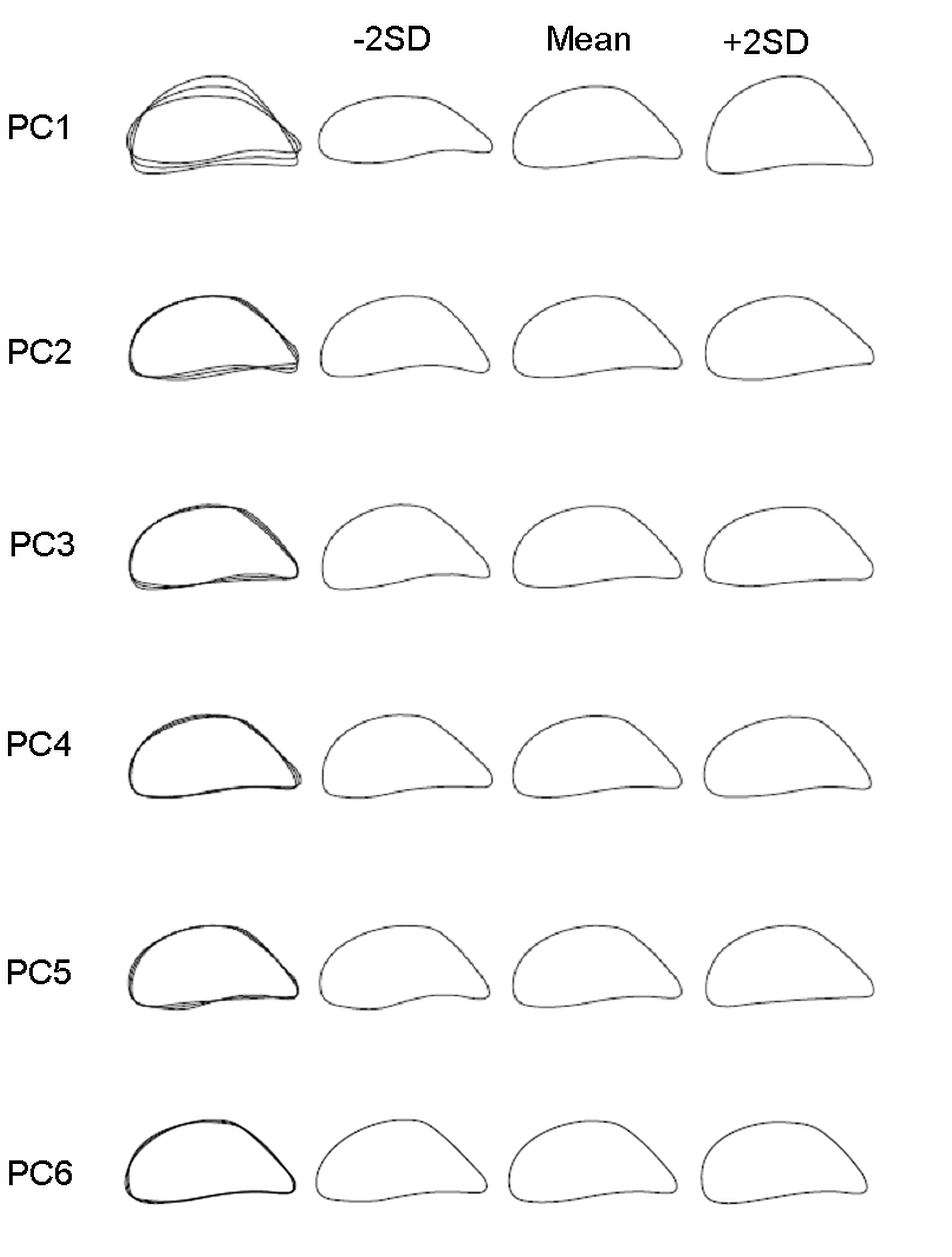

Supplement: S6 Figure — Visualization of contour shapes. Contour shapes are visualized as the 6 effective components from the elliptic Fourrier analysis in SHAPE v 1.3 explaining 99.8% of the variation in the total data. (TIF) [file pone.0115192.s006.tif]
