# Supplementary material for: Plastic Responses of a Sessile Prey to Multiple Predators: A Field and Experimental Study
Source: PLoS One. 2014 Dec 17;9(12):e115192. doi: 10.1371/journal.pone.0115192 (PMC4269437; doi:10.1371/journal.pone.0115192)
Supplement: S1 Table — Raw data for PLS model. Data on factors tested in the PLS to explain zebra mussel phenotypes in the field. (PDF) [file pone.0115192.s007.pdf]

Table S1. Raw data for PLS model. Data on factors tested in the PLS to explain zebra mussel phenotypes in the field

Mussel density	% bottom stone covered	% soft bottom	% bottom macrophyte covered	Chl-a concentration µg/L	Wave exposure (km)	Shell strength	PC1 shape	PC2 shape	Crayfish CPUE g/traps	non-roach cyprinid population density CPUE g/nets	Population density roach CPUE g/nets
40.70	69.19	22.06	8.75	441.57	10.42	0.01	-0.03	0.00	1026.40	0.00	1292.90
39.63	3.63	94.90	1.47	1801.34	16.27	0.02	-0.03	0.00	118.00	1172.00	7590.60
3.86	16.03	19.68	66.31	163.30	16.56	-0.18	-0.01	-0.01	1094.50	1467.00	4515.60
13.59	44.57	0.00	56.16	61.84	15.72	0.16	-0.03	0.00	NA	7240.40	7022.60
13.20	7.37	0.00	92.17	66.39	6.29	-0.18	-0.05	-0.01	1565.00	1306.20	4671.30
0.93	16.92	13.33	69.74	56.62	2.50	-0.21	-0.03	0.00	1039.06	2282.60	5010.70
92.13	80.81	0.00	19.19	117.27	11.76	0.26	-0.04	0.01	1572.00	44.30	1955.80
17.23	4.01	92.98	3.01	368.38	7.93	-0.28	-0.03	0.01	2956.70	115.20	1240.90
69.64	57.01	7.94	35.05	306.31	4.10	0.40	-0.03	0.00	906.83	1603.00	5276.10
