# Supplementary material for: Plastic Responses of a Sessile Prey to Multiple Predators: A Field and Experimental Study
Source: PLoS One. 2014 Dec 17;9(12):e115192. doi: 10.1371/journal.pone.0115192 (PMC4269437; doi:10.1371/journal.pone.0115192)
Supplement: S2 Table — Raw data for mussel field data. Data on shell shape and strength and size of zebra mussels in the field. (PDF) [file pone.0115192.s008.pdf]

Table S2. Raw data for mussel field data. Data on shell shape and strength and size of zebra mussels in the field

Site	PC1	PC2	growth	shell strength	height	length	width
site 1	-0.0926	0.0228	15.88	-0.2594	14.71	28.85	12.22
site 1	-0.0846	0.0311	13.90	-0.0920	12.11	25.65	11.07
site 1	-0.1008	-0.0115		0.3171	11.20	22.42	9.27
site 1	0.0543	0.0027	10.99	0.2071	10.58	19.05	10.23
site 1	-0.1155	-0.0062	15.92	0.1391	12.90	26.53	10.07
site 1	0.0045	0.0314	12.71	0.3008	11.24	22.05	10.97
site 1	-0.0827	0.0169	15.78	-0.1508	13.14	28.43	12.48
site 1	0.0285	0.0113	13.06	-0.0228	10.95	22.83	11.85
site 1	-0.0622	0.0135		-0.7080	14.48	27.82	12.52
site 1	-0.0388	-0.0033	11.68	-0.0052	12.79	23.64	10.89
site 1	-0.0093	-0.0046	14.98	0.1162	12.51	22.41	11.34
site 1	-0.0020	0.0048	13.02	0.0571	13.59	24.25	12.18
site 1	-0.0587	0.0169	16.48	-0.0073	13.15	22.30	10.37
site 1	-0.0527	-0.0025		0.1617	10.68	20.60	9.60
site 1	-0.0096	-0.0040	11.24	0.3534	11.93	22.03	10.63
site 1	-0.0128	0.0141		-0.3261	13.97	25.82	12.30
site 1	-0.0590	-0.0050	17.50	0.0906	11.57	23.32	10.02
site 1	-0.0780	0.0070	15.06	0.0371	12.83	24.98	10.80
site 1	-0.1075	0.0210	19.80	-0.0213	11.39	26.47	10.64
site 1	-0.0883	0.0159	12.63	0.2166	11.41	24.58	10.31
site 1	0.0269	-0.0108	15.27	0.0317	12.06	21.56	11.56
site 1	0.0302	-0.0157	11.87	0.5062	10.93	19.29	10.17
site 1	-0.0896	0.0249	16.02	0.0606	11.48	22.40	9.81
site 1	-0.0337	0.0042	18.09	0.0747	12.96	25.10	11.45
site 1	-0.0515	0.0190	14.47	-0.6550	17.36	24.49	11.19
site 1	0.0153	0.0024	8.45	0.4134	11.33	19.98	10.00
site 1	-0.0774	0.0024	16.11	-0.1089	12.73	24.81	10.96
site 1	-0.0194	0.0106	11.72	0.6473	12.72	25.17	12.00
site 1	0.0163	0.0145	11.43	0.0944	12.47	23.56	12.55
site 1	-0.0469	-0.0221	14.41	0.5496	13.28	25.34	11.58
site 1	-0.0724	0.0082	13.66	0.4738	11.29	23.94	10.22
site 1	0.0423	0.0265	12.53	0.1830	11.42	21.92	12.03
site 1	-0.0565	0.0226	19.62	-0.6390	15.30	28.21	12.55
site 1	-0.0417	-0.0013	12.86	0.4299	11.75	23.37	10.70
site 1	-0.0010	-0.0212	12.99	0.4032	11.72	20.72	10.71
site 1	-0.0522	0.0159	18.25	-0.2084	12.47	24.47	11.10
site 1	-0.0747	0.0118	14.86	-0.0216	14.18	26.67	11.89
site 1	-0.0110	0.0201	14.38	0.2959	13.66	25.54	12.27
site 1	-0.0528	-0.0032	13.97	-0.2388	14.50	26.97	12.26
site 1	-0.0006	-0.0334	9.60	0.1251	10.92	21.51	10.88
site 1	0.0059	-0.0121	13.63	0.1225	13.37	25.08	12.30
site 1	0.0059	-0.0061	14.08	-0.3918	14.78	26.67	13.53

site 1	0.0031	-0.0077	14.70	0.1018	14.20	24.61	12.40
site 1	-0.0362	-0.0051	13.18	0.3778	11.48	23.96	11.36
site 1	-0.0727	-0.0121	13.37	-0.3209	12.72	25.01	11.38
site 1	-0.0818	-0.0194	20.10	-0.2192	13.38	25.95	11.45
site 1	-0.0260	0.0043	16.79	-0.0590	13.55	22.72	10.69
site 1	-0.0451	0.0428	15.57	-0.0332	11.92	22.82	11.26
site 1	-0.0063	0.0220	15.27	-0.5277	14.34	26.92	12.99
site 1	-0.1144	0.0343	14.81	0.3331	11.43	24.07	10.22
site 1	-0.0378	-0.0026		0.0096	11.89	22.51	10.15
site 1	-0.0549	0.0066	15.70	-0.0481	12.75	24.24	11.29
site 1	0.0402	-0.0352		0.3720	15.58	27.14	13.94
site 1	-0.0181	0.0198	12.84	-0.3973	14.77	26.89	13.00
site 1	-0.0884	-0.0368	22.71	-0.5666	16.50	30.74	12.58
site 1	-0.0154	0.0153	12.18	-0.1656	12.04	22.28	11.98
site 1	-0.0122	-0.0203	10.69	0.4914	11.84	22.10	10.53
site 1	0.0009	-0.0079	12.70	0.1276	13.14	24.77	12.02
site 1	-0.0132	-0.0179	16.20	0.0524	15.05	27.84	13.08
site 1	0.0584	-0.0164	9.23	0.0685	12.52	22.02	11.97
site 1	-0.0123	-0.0219	13.43	-0.5265	13.14	25.20	12.85
site 1	0.0342	-0.0261		0.5456	12.35	22.49	11.27
site 1	-0.0955	0.0006	19.66	-0.3341	12.33	26.52	11.05
site 1	-0.0026	-0.0039	13.29	-0.2581	12.68	24.38	11.96
site 1	-0.0460	0.0024	14.41	-0.3622	12.68	27.27	12.24
site 1	-0.0228	0.0065		0.1844	11.77	21.03	10.31
site 1	0.0014	-0.0019	12.63	-1.1387	15.61	26.94	17.82
site 1	-0.0586	0.0002	14.11	-0.1910	12.73	23.90	10.45
site 1	0.0031	-0.0043	15.32	0.2571	12.53	25.36	13.30
site 1	-0.0481	0.0311	12.13	-0.1297	13.74	26.50	12.12
site 1	0.0086	-0.0005	11.20	0.1535	13.38	23.85	12.10
site 1	0.0288	-0.0108	11.90	-0.0741	12.24	20.80	11.01
site 1	0.0146	-0.0090	11.67	0.1150	12.18	22.17	11.21
site 1	0.0365	-0.0195	14.28	0.0885	12.86	24.30	12.68
site 1	-0.0259	0.0064	21.60	0.1499	13.20	26.58	12.68
site 1	-0.0510	-0.0188		0.1404	11.63	22.14	9.84
site 1	-0.0647	0.0097		-0.2623	13.21	25.22	11.09
site 1	-0.0230	0.0182	15.38	0.0837	12.51	25.80	12.25
site 1	-0.0259	-0.0185		-0.1585	12.37	24.47	10.98
site 1	-0.0214	0.0068		-0.4173	13.00	25.47	12.12
site 1	-0.0532	-0.0338		0.0164	11.84	21.47	10.03
site 1	-0.0463	-0.0249		-0.1748	12.10	23.97	11.05
site 1	-0.0574	0.0134	15.12	0.3663	12.12	23.31	10.22
site 1	-0.0252	0.0081	15.09	0.7076	12.69	22.54	10.69
site 2	-0.0614	0.0125	17.63	-0.2344	12.05	24.94	10.66
site 2	-0.0363	-0.0055	12.72	-0.1765	12.05	22.20	10.38
site 2	-0.0293	0.0051		-0.1858	11.20	23.33	10.92
site 2	-0.0233	-0.0167	33.46	-0.5057	12.76	27.20	12.15

site 2	-0.0483	0.0221	12.78	0.1213	11.93	22.51	9.87
site 2	-0.0648	0.0047		-0.0331	11.97	23.69	10.26
site 2	-0.0259	0.0112		-0.1854	11.93	22.74	10.91
site 2	-0.0400	0.0062	11.51	-0.4055	13.17	24.41	11.01
site 2	-0.0092	0.0071	12.71	-0.2170	12.09	22.73	10.63
site 2	-0.0337	0.0087		-0.2940	11.78	24.31	11.07
site 2	-0.0845	0.0109	17.04	0.0745	11.70	21.82	9.62
site 2	0.0010	0.0037	14.17	0.0741	10.80	21.48	10.50
site 2	-0.0449	0.0133	13.91	0.0135	11.56	20.98	9.59
site 2	-0.0095	0.0298	9.41	0.1153	11.15	19.42	9.37
site 2	-0.0714	0.0003	13.06	-0.0110	10.42	21.90	9.54
site 2	-0.0329	-0.0178		-0.0957	11.03	23.17	10.96
site 2	-0.0367	0.0090	13.98	-0.3585	12.16	23.41	10.85
site 2	-0.0319	-0.0045	13.86	0.0888	11.81	21.12	9.61
site 2	-0.0625	0.0259	15.21	0.0586	11.01	20.58	9.68
site 2	-0.0372	0.0085	12.66	0.2961	9.25	19.55	8.76
site 2	-0.0407	-0.0153	11.84	0.2443	10.64	19.44	9.00
site 2	0.0104	-0.0293		0.1051	10.19	19.51	10.10
site 2	-0.0713	0.0229	12.17	-0.0303	11.07	21.75	10.07
site 2	-0.0328	-0.0242		0.2265	11.48	20.95	9.60
site 2	-0.0465	-0.0080	14.44	0.0399	11.06	21.57	9.73
site 2	-0.0115	0.0009	12.70	0.0650	10.71	20.91	9.71
site 2	0.0300	-0.0169	13.31	-0.2636	12.34	22.16	11.49
site 2	0.0696	-0.0187	12.04	-0.0986	11.53	19.91	11.11
site 2	-0.0486	-0.0080	12.94	-0.1847	12.50	23.91	10.17
site 2	-0.1028	0.0322	12.31	0.0583	9.75	22.15	9.82
site 2	-0.0019	-0.0029		-0.1013	11.40	22.31	10.80
site 2	-0.0921	0.0219		-0.1757	12.26	22.32	9.72
site 2	-0.0435	0.0108	12.80	0.1249	10.82	21.46	9.60
site 2	0.0195	-0.0139	11.04	0.0100	12.10	19.52	10.13
site 2	-0.0402	0.0036		0.4162	10.74	18.65	8.38
site 2	-0.0446	-0.0065	10.49	0.1517	9.89	21.08	9.59
site 2	-0.0507	-0.0071	11.20	0.0737	10.59	21.80	9.64
site 2	-0.0579	0.0109	14.02	-0.2599	11.25	24.41	10.93
site 2	0.0015	0.0092	10.97	0.0885	10.77	20.63	9.97
site 2	-0.0166	0.0124		-0.2807	13.30	24.00	11.36
site 2	0.0149	-0.0180	13.58	-0.1597	12.10	22.26	11.35
site 2	-0.0616	-0.0304	13.51	0.1261	10.75	21.74	9.77
site 2	-0.0289	-0.0213		-0.1292	12.99	21.20	10.02
site 2	-0.0198	-0.0035		-0.0078	10.72	21.37	10.15
site 2	-0.0164	-0.0160	29.49	0.1259	10.81	20.00	9.68
site 2	0.0105	0.0090	19.08	-0.2266	11.47	22.08	10.92
site 2	-0.0290	0.0012	12.34	0.0836	11.18	21.93	10.33
site 2	-0.0479	-0.0162	12.43	0.3421	10.39	18.68	8.50
site 2	-0.0228	-0.0039		0.0441	10.49	21.14	10.21
site 2	-0.0005	-0.0123	10.61	0.5169	9.38	17.01	8.48

site 2	0.0079	0.0186	13.88	0.0890	11.17	20.92	10.72
site 2	-0.0596	-0.0046		0.2707	10.67	19.85	9.11
site 2	0.0063	-0.0078		0.4118	9.89	16.97	9.09
site 2	-0.0451	0.0274	11.34	0.1151	11.35	19.13	8.90
site 2	0.0091	0.0051	12.96	0.0087	11.41	19.35	9.85
site 2	-0.0182	-0.0190	12.68	0.2977	10.22	17.76	9.02
site 2	-0.0221	-0.0171	13.25	0.1383	10.65	20.13	9.39
site 2	-0.0548	0.0018	14.18	0.3154	10.49	19.86	8.65
site 2	0.0032	0.0175	11.49	-0.0441	10.82	21.04	10.34
site 2	-0.0539	-0.0026	12.28	0.1696	10.46	21.42	9.15
site 2	-0.0139	-0.0136	13.62	-0.0469	11.31	20.65	10.11
site 2	-0.0162	-0.0184		0.2611	10.73	18.75	8.88
site 2	-0.0778	0.0068	14.98	-0.0099	10.98	22.38	9.64
site 2	0.0170	-0.0293	12.77	0.0117	11.36	20.58	10.47
site 2	-0.0992	-0.0056	11.40	0.5757	10.35	17.77	7.54
site 2	-0.0361	-0.0197	12.28	0.2709	11.38	18.76	8.69
site 2	-0.0432	-0.0147	13.79	0.0242	10.86	20.54	9.77
site 2	-0.0099	-0.0018	12.76	0.0935	12.03	21.56	10.15
site 2	0.0080	0.0183	14.13	-0.2008	11.43	20.88	10.84
site 2	-0.0120	-0.0039	11.58	0.0437	10.12	20.45	10.00
site 2	-0.0390	-0.0140	14.05	0.0116	10.60	20.91	9.97
site 2	-0.0218	0.0073		0.1834	10.59	18.44	9.56
site 2	-0.0603	0.0027	12.34	-0.0169	11.52	21.67	9.94
site 2	0.0079	-0.0048	13.81	-0.0033	11.02	20.30	10.29
site 2	-0.0993	-0.0086		-0.1129	12.36	24.14	9.74
site 2	-0.0167	-0.0228		-0.2797	12.19	22.94	10.97
site 2	-0.0097	0.0003	11.90	0.0894	11.18	20.01	9.62
site 2	-0.0396	0.0083	11.65	-0.5120	13.32	24.67	11.58
site 2	-0.0649	0.0053	13.70	0.1160	10.65	20.96	9.43
site 2	-0.0206	-0.0159		-0.1093	12.14	23.37	11.04
site 2	-0.0126	0.0249	11.44	-0.0038	11.07	20.66	10.30
site 2	-0.0178	-0.0204	13.02	-0.2859	12.70	22.58	10.94
site 2	-0.0025	-0.0028		0.5522	10.01	17.98	8.95
site 2	-0.0233	0.0075	8.71	0.3732	11.05	18.58	8.90
site 3	0.0024	-0.0079	19.19	-0.7580	16.89	30.49	15.02
site 3	-0.0763	0.0117	19.87	-1.5019	20.16	32.87	14.32
site 3	-0.0117	-0.0149	16.62	-0.7481	15.59	28.19	14.24
site 3	-0.0171	0.0100	22.74	-1.4955	20.79	32.96	16.80
site 3	-0.0208	0.0007	16.73	-0.5187	16.62	30.19	14.22
site 3	-0.0370	-0.0036	18.93	-1.1843	19.08	32.96	15.44
site 3	-0.0185	-0.0207	15.43	-0.8640	14.95	27.68	13.26
site 3	-0.0202	0.0131	14.27	-0.4198	12.85	25.18	11.74
site 3	-0.0242	-0.0061		1.5672	17.52	29.15	13.64
site 3	-0.0058	-0.0017		-1.0068	16.18	30.59	14.32
site 3	0.0088	-0.0045	15.17	-0.2886	14.27	26.50	12.78
site 3	0.0072	0.0005	16.42	-0.2524	10.38	23.29	12.00

site 3	-0.0185	0.0065	12.56	-0.9464	16.41	27.49	13.21
site 3	-0.0145	-0.0075	12.45	-0.3202	12.81	25.12	11.80
site 3	0.0101	-0.0094	16.17	-0.5244	13.30	23.25	12.31
site 3	-0.0202	-0.0052	16.82	-0.1308	12.00	25.73	12.30
site 3	-0.0259	-0.0214	10.72	-0.5363	13.87	25.24	12.76
site 3	-0.0810	-0.0021	16.91	-0.5387	13.96	27.01	11.22
site 3	0.0025	-0.0147		-0.7898	15.06	29.80	14.57
site 3	0.0031	0.0079	12.17	-0.1568	10.27	22.91	10.71
site 3	-0.0645	-0.0316	15.24	-0.3627	14.84	28.39	12.74
site 3	-0.0293	-0.0312	12.41	-1.0186	14.65	30.12	14.16
site 3	0.0109	-0.0051	17.16	-0.1791	12.04	22.40	11.31
site 3	-0.0014	-0.0077	15.93	0.1834	10.31	20.27	9.78
site 3	0.0024	-0.0130	15.34	-0.5653	13.72	25.18	12.27
site 3	-0.0049	-0.0151	11.96	-0.6526	15.20	26.82	13.24
site 3	0.0185	0.0153	11.76	-0.3796	12.63	24.56	12.80
site 3	-0.0565	0.0056	14.81	0.0100	12.89	27.44	12.21
site 3	-0.0169	-0.0006	16.37	-0.2568	13.02	24.50	11.64
site 3	-0.0212	-0.0002	10.75	-0.4363	15.30	28.53	13.82
site 3	-0.0007	-0.0054	16.24	-0.4016	13.02	25.08	11.64
site 3	-0.0367	-0.0170		-0.1992	12.47	25.97	12.20
site 3	-0.0017	-0.0021	13.05	-0.7438	14.72	27.29	12.98
site 3	-0.0150	0.0024	10.90	-0.1837	11.50	22.46	10.75
site 3	0.0019	-0.0200		-0.1874	11.95	23.84	11.97
site 3	0.0323	-0.0110	18.57	0.1829	9.53	19.35	9.98
site 3	-0.0065	0.0173	16.08	-0.2766	11.68	23.33	10.72
site 3	0.0178	0.0070	20.73	-0.1903	11.88	22.54	11.52
site 3	-0.0084	0.0147	12.43	0.1362	11.40	21.06	9.97
site 3	0.0714	0.0035	14.65	0.0695	10.43	19.56	10.31
site 3	-0.0132	-0.0283	17.76	0.1379	14.26	27.05	12.57
site 3	-0.0488	-0.0184	13.82	-0.0522	15.70	28.38	12.84
site 3	-0.0416	0.0092	13.65	-0.6149	16.08	28.39	14.18
site 3	-0.0114	-0.0103	9.78	-0.5227	12.82	26.57	12.77
site 3	0.0064	-0.0100	11.67	0.1473	10.00	19.17	9.72
site 3	-0.0052	0.0109	9.22	0.1699	9.52	19.85	9.84
site 3	-0.0234	-0.0291	10.84	0.2371	10.57	19.88	9.52
site 3	-0.0227	0.0012	11.06	-0.3477	12.44	23.90	11.17
site 3	-0.0348	-0.0034	9.40	-0.7597	13.90	28.67	13.35
site 3	0.0111	-0.0098	10.25	-0.4889	15.30	27.42	14.02
site 3	-0.0179	-0.0074	15.40	0.0321	10.88	21.39	10.16
site 3	-0.0858	-0.0319	21.07	-0.0937	12.69	19.77	10.74
site 3	-0.0303	-0.0204	15.23	-0.7765	14.30	26.80	12.91
site 3	0.0012	-0.0101	13.68	0.2578	10.02	20.44	9.81
site 3	0.0689	-0.0159		0.5160	8.30	16.70	8.70
site 3	-0.0133	0.0208	9.60	-0.0344	10.93	21.24	10.43
site 3	0.0918	-0.0160	10.46	0.6034	8.56	15.82	9.04
site 3	-0.0270	-0.0206	11.46	-0.6538	15.17	24.52	11.94

site 3	-0.0169	-0.0005	14.14	-0.5181	12.38	25.53	11.93
site 3	-0.0267	0.0272	11.96	-1.2780	17.79	31.11	14.54
site 3	0.0187	-0.0158	9.36	-0.2430	12.12	22.36	11.34
site 3	0.0866	-0.0101	17.14	1.0501	6.10	13.40	7.49
site 3	0.0283	-0.0373	15.82	0.4734	8.64	16.46	9.02
site 3	-0.0186	-0.0028	10.22	-0.3392	12.87	25.10	12.67
site 3	0.0030	-0.0286	9.24	0.1905	9.86	19.06	9.54
site 3	-0.0191	0.0067	11.23	0.3753	8.69	18.85	8.93
site 3	0.0456	-0.0352		0.4864	8.55	16.51	9.05
site 3	0.0244	-0.0131	13.11	-0.6245	15.20	29.93	15.90
site 3	0.0040	0.0136	13.03	0.5731	8.72	17.30	8.31
site 3	-0.0078	-0.0031		0.3002	10.68	22.56	10.00
site 3	-0.0016	0.0028		0.0531	11.43	20.41	9.63
site 3	-0.0367	-0.0126	7.30	0.2789	10.50	20.95	9.83
site 3	-0.0088	-0.0237	9.79	-0.0515	11.91	21.31	10.35
site 3	0.0199	0.0130	16.66	0.1309	11.42	21.52	10.79
site 3	-0.0469	-0.0227	9.91	0.0734	10.79	21.40	9.76
site 3	-0.0396	-0.0052	9.52	0.0675	10.96	22.69	10.61
site 3	-0.0716	-0.0333	9.19	0.6067	8.64	16.75	7.52
site 3	0.0208	-0.0278		0.2799	10.75	19.00	10.02
site 3	0.0220	0.0035		-0.1303	12.77	21.89	11.33
site 3	-0.0212	-0.0055	13.38	0.6048	9.68	18.18	8.65
site 3	-0.0477	-0.0067	12.03	0.5149	9.60	19.72	8.80
site 3	-0.0550	0.0045		0.1914	12.04	22.44	9.59
site 3	-0.0493	-0.0057		0.6003	12.30	22.82	10.24
site 3	0.0070	-0.0225	12.57	0.3655	12.20	19.63	9.52
site 4	-0.0387	0.0011		-0.1783	12.29	22.53	10.32
site 4	0.0158	-0.0029		0.4423	9.84	17.28	9.07
site 4	-0.0026	0.0011		-0.1821	14.07	22.95	11.73
site 4	-0.0336	-0.0050	12.30	0.1030	11.35	20.40	9.50
site 4	-0.0600	0.0263	9.64	0.2907	10.46	19.80	8.72
site 4	-0.0385	-0.0084	13.96	-0.1677	11.67	22.70	10.18
site 4	-0.0570	-0.0159	12.64	-0.2852	13.58	22.71	9.67
site 4	-0.0427	0.0434	10.65	-0.0358	11.10	21.48	10.26
site 4	-0.0019	0.0073		0.1134	11.53	19.52	9.45
site 4	-0.0358	0.0061		0.4633	9.14	18.02	9.05
site 4	0.0305	-0.0077	8.11	0.1998	10.73	18.03	9.68
site 4	-0.0145	-0.0262	9.91	0.2709	10.29	18.83	9.24
site 4	0.0001	0.0289	7.51	-0.0151	11.22	20.90	10.48
site 4	-0.0328	-0.0055	8.02	0.3361	10.00	18.18	8.52
site 4	-0.0634	0.0121		0.5551	9.30	17.66	7.63
site 4	-0.0569	-0.0120	12.24	0.3443	12.78	22.80	10.23
site 4	-0.0029	0.0017		0.3519	10.25	19.65	9.78
site 4	-0.0007	-0.0226	9.14	0.1192	10.33	20.61	10.04
site 4	-0.0268	0.0027	9.93	0.2276	10.46	19.60	8.97
site 4	-0.0548	0.0275	11.49	0.2125	10.97	20.62	9.02

site 4	0.0003	-0.0330	10.84	-0.2222	11.91	22.35	10.63
site 4	-0.0639	-0.0164	11.42	-0.0962	12.57	21.55	9.70
site 4	-0.0992	-0.0102		0.3425	11.79	22.53	9.29
site 4	0.0320	-0.0185		0.1045	11.57	18.72	10.43
site 4	-0.0643	0.0086	9.18	0.3876	9.83	18.92	8.37
site 4	-0.0466	-0.0009	10.97	0.2867	10.13	20.55	9.03
site 4	-0.0708	-0.0026		0.1248	12.18	22.40	9.45
site 4	-0.0032	0.0284	8.43	0.5297	9.85	16.76	7.92
site 4	-0.0047	0.0100		0.7113	8.93	16.14	7.52
site 4	-0.0320	0.0075	12.75	-0.2836	13.00	23.49	11.24
site 4	-0.1107	0.0028	16.30	-0.3021	13.79	25.45	11.20
site 4	-0.0729	-0.0127	13.80	-0.4823	15.55	26.52	11.94
site 4	-0.0436	-0.0035	12.46	-0.2117	11.98	23.37	10.64
site 4	-0.1187	0.0089	14.99	-0.6180	15.01	27.05	11.06
site 4	-0.0836	0.0153	13.62	-0.2199	14.55	25.60	11.31
site 4	-0.0657	-0.0034		-0.1170	12.79	26.23	11.76
site 4	-0.0542	0.0060	11.41	-0.1450	13.00	25.35	11.83
site 4	-0.0484	0.0239	9.98	-0.0128	11.40	22.36	10.39
site 4	-0.1040	0.0186	11.65	-0.3454	12.96	24.50	10.50
site 4	-0.0285	-0.0078	8.35	-0.2254	12.63	23.15	10.51
site 4	-0.0664	0.0290	13.42	-0.1037	13.30	26.30	11.57
site 4	-0.0198	-0.0180	11.45	0.2778	11.43	20.16	11.54
site 4	0.0084	-0.0163		0.1276	11.54	19.67	10.04
site 4	0.0335	0.0204		0.4579	11.00	20.00	10.34
site 4	-0.0662	0.0148	13.92	0.1502	11.46	23.91	10.21
site 4	-0.0270	0.0029		0.3243	11.42	21.68	10.34
site 4	0.0040	0.0037	8.97	-0.0259	12.00	19.90	9.82
site 4	-0.0492	-0.0046	12.92	0.5487	13.53	20.93	9.34
site 4	-0.0094	-0.0030	10.97	0.1882	12.28	21.27	10.23
site 4	-0.0375	0.0119	10.81	0.3202	11.09	19.63	8.94
site 4	0.0636	-0.0143	10.20	0.2959	9.75	17.40	9.94
site 4	-0.0425	-0.0051	14.69	-0.1887	13.64	24.05	11.35
site 4	-0.0590	-0.0003		0.5631	11.98	20.53	9.08
site 4	-0.0451	0.0034		0.0269	12.00	22.08	9.90
site 4	-0.0380	-0.0252	9.30	0.4935	10.60	18.12	8.52
site 4	-0.0401	-0.0236	9.90	0.2982	11.92	21.07	9.91
site 4	-0.0253	0.0284	10.23	-0.0135	11.35	21.12	9.83
site 4	-0.0227	0.0156	11.20	0.0969	11.53	20.48	9.58
site 4	-0.0591	0.0172	9.79	0.0694	12.06	22.02	9.75
site 4	0.0195	0.0043	8.21	0.5411	9.57	17.28	8.40
site 4	-0.0605	0.0066	9.95	0.3541	10.88	19.32	8.39
site 4	0.0104	0.0183	8.45	0.3496	10.59	18.30	8.69
site 4	-0.0090	0.0235	9.76	0.6173	10.15	17.65	8.36
site 4	-0.0117	-0.0233	10.61	0.2525	10.73	20.02	10.04
site 4	-0.0600	-0.0114	14.58	-0.0984	12.00	22.26	9.80
site 4	0.0118	-0.0120	10.40	0.5164	10.82	19.72	9.93

site 4	-0.0076	0.0099	9.05	0.6558	9.44	16.72	7.87
site 4	-0.0518	0.0373	11.33	-0.4137	13.53	23.95	10.71
site 4	-0.0020	-0.0040	8.49	0.4235	9.95	17.25	8.23
site 4	-0.0630	-0.0031	9.12	1.0776	9.62	18.51	8.26
site 4	-0.0487	-0.0199	11.36	0.3656	12.82	22.73	10.78
site 4	-0.0440	-0.0428	9.12	0.7053	9.95	17.84	7.75
site 4	-0.0548	0.0008		0.0248	11.44	22.10	9.99
site 4	-0.0640	-0.0042		-0.0400	11.39	23.37	10.01
site 4	-0.0164	-0.0273	11.14	-0.2366	12.76	23.62	10.95
site 4	-0.0079	0.0098	11.82	0.0285	11.96	21.54	10.54
site 4	0.0043	-0.0079		0.1542	11.48	19.65	9.76
site 4	0.0223	-0.0195	9.94	0.2227	11.05	20.14	9.96
site 4	-0.0362	0.0000	8.68	0.2905	9.74	19.42	8.74
site 4	0.0057	0.0094		0.4060	10.20	18.84	9.34
site 4	0.0076	0.0045	8.89	0.3428	9.94	18.21	8.74
site 4	0.0085	0.0154	9.38	0.4115	10.66	18.86	9.82
site 4	-0.0179	-0.0232	12.16	0.4058	9.80	19.56	9.19
site 4	-0.0600	0.0050		0.2177	11.32	20.00	8.53
site 5	-0.1076	-0.0565	22.71	-0.9867	18.85	35.14	13.58
site 5	-0.0597	0.0274	20.96	-1.2823	18.61	32.85	14.15
site 5	-0.0875	-0.0538	19.69	-0.5799	18.37	33.02	14.25
site 5	-0.0815	-0.0021	20.14	-0.7644	18.25	33.00	14.01
site 5	-0.0470	0.0093	15.27	-0.7421	19.05	30.50	13.78
site 5	-0.1122	0.0065	16.95	-0.2319	16.28	31.12	13.02
site 5	-0.0442	-0.0098	15.50	0.3942	14.93	28.03	12.33
site 5	-0.0568	-0.0210		0.8003	15.59	28.58	11.99
site 5	-0.0193	0.0092	14.97	-0.6908	17.38	30.63	14.29
site 5	-0.1059	-0.0260		-1.0950	19.34	35.09	13.84
site 5	-0.0816	-0.0054	14.58	-0.5881	18.57	29.85	12.27
site 5	-0.0700	0.0050	14.20	0.2867	15.54	26.14	12.05
site 5	-0.0830	0.0179	17.06	0.0177	15.36	32.60	13.88
site 5	-0.1146	0.0166	15.95	-0.3649	16.35	34.03	13.74
site 5	-0.0498	0.0113	12.27	0.2929	14.73	26.95	12.46
site 5	0.0114	-0.0169	14.46	0.1308	15.61	28.40	14.53
site 5	-0.0185	-0.0369	14.81	-0.5807	16.82	29.89	14.40
site 5	-0.0332	-0.0327	15.63	-0.2290	15.27	28.39	13.84
site 5	-0.0587	-0.0120	15.39	-0.2064	19.24	29.12	13.57
site 5	-0.0800	-0.0173	16.03	-0.1878	18.05	29.55	12.45
site 5	-0.0201	-0.0245	18.99	0.6461	16.81	28.65	13.68
site 5	-0.0547	-0.0559	21.14	-0.4746	18.76	32.85	15.16
site 5	-0.0230	-0.0407	16.27	0.7782	15.85	26.45	12.65
site 5	-0.0983	-0.0509	21.46	0.2523	17.95	35.51	13.82
site 5	-0.1511	-0.0124	15.50	0.1015	16.45	32.33	11.64
site 5	-0.0561	-0.0151	20.24	0.1609	19.06	30.94	13.52
site 5	-0.1087	-0.0251	15.94	0.9365	16.95	27.91	11.27
site 5	0.0300	-0.0052	14.72	0.3904	16.38	28.28	15.59

site 5	-0.0407	-0.0052	15.67	0.0323	15.72	30.52	13.38
site 5	-0.0830	-0.0229	16.79	0.3059	17.04	33.46	13.53
site 5	-0.0737	-0.0109	18.00	0.0025	16.55	32.41	14.13
site 5	-0.0854	-0.0437	20.34	-0.5377	18.08	35.97	15.15
site 5	-0.0421	-0.0203	18.39	-0.1163	17.28	31.47	13.65
site 5	0.0525	-0.0265	11.69	0.2747	15.99	23.63	13.23
site 5	0.0062	-0.0016	14.20	-0.2114	16.92	29.21	14.94
site 5	-0.0178	0.0096	13.85	-0.6392	13.17	28.87	12.85
site 5	0.0110	0.0142	12.46	-0.3685	15.26	27.87	13.63
site 5	-0.0194	-0.0214	16.07	0.3909	15.00	30.85	14.60
site 5	-0.0599	-0.0008	13.90	0.2473	15.60	27.29	15.90
site 5	-0.0390	-0.0110	14.55	-0.5065	16.10	30.04	13.93
site 5	-0.0020	-0.0168	15.39	0.5076	17.05	28.62	14.54
site 5	-0.0418	-0.0001	19.71	-0.3544	17.92	33.16	15.29
site 5	-0.0404	-0.0194	16.92	0.0079	16.04	30.56	13.78
site 5	-0.0474	-0.0162	21.55	0.8177	18.06	31.53	14.27
site 5	-0.0769	-0.0006	18.73	0.3018	19.87	34.67	13.91
site 5	-0.0625	-0.0194		-0.2849	18.27	30.17	13.42
site 5	-0.1072	-0.0156	20.98	-0.5904	17.39	34.79	13.59
site 5	-0.0717	-0.0369		-1.1358	18.61	30.40	13.11
site 5	-0.1001	-0.0353	14.65	-0.5659	14.62	27.06	10.89
site 5	-0.0057	-0.0244	15.17	-0.1988	17.61	29.64	14.27
site 5	-0.0095	-0.0704	16.24	-0.2325	14.81	26.49	13.75
site 5	-0.0481	-0.0371	14.45	-0.1680	15.10	25.28	11.85
site 5	-0.1021	0.0004	16.26	-0.3493	17.18	28.91	11.66
site 5	-0.0544	0.0000	21.41	-0.4282	18.52	35.05	15.52
site 5	-0.0676	-0.0128	16.62	-1.3808	18.37	32.52	15.00
site 5	-0.0564	-0.0141	17.65	-0.2239	17.45	30.63	13.65
site 5	0.0240	-0.0013	14.54	-0.5035	18.72	29.21	14.93
site 5	-0.0896	0.0017	18.57	0.0319	17.87	31.22	13.13
site 5	-0.0104	-0.0248	17.47	-0.3290	17.19	31.05	15.09
site 5	-0.0267	-0.0362	16.66	-0.2101	17.72	28.71	14.24
site 5	-0.0261	-0.0086	15.67	-0.1037	16.73	28.28	13.69
site 5	-0.0908	0.0086	14.59	0.0619	16.72	30.02	12.82
site 5	0.0509	0.0045	11.18	-0.3388	14.36	22.43	11.74
site 5	-0.0653	-0.0001	15.63	-0.1759	16.93	29.36	12.88
site 5	-0.0485	-0.0270	18.61	-0.7174	17.77	32.45	14.34
site 5	-0.0973	0.0020	19.73	-0.6149	14.70	33.08	13.62
site 5	-0.0394	-0.0405	16.35	-0.7331	18.68	30.62	14.52
site 5	-0.1171	-0.0701	19.26	0.6930	17.08	30.31	12.55
site 5	0.0233	-0.0451	15.02	-1.1095	16.32	28.64	14.97
site 5	-0.0698	-0.0377	19.20	-0.2767	19.01	30.16	13.48
site 5	-0.0940	0.0438		-0.6267	17.01	29.88	12.55
site 5	-0.0042	-0.0049		0.0463	15.91	28.71	13.54
site 5	-0.1235	-0.0108	20.00	0.1549	17.27	33.28	13.44
site 5	-0.0937	-0.0199	20.80	1.4077	19.04	34.62	13.98

site 5	-0.0740	-0.0447	19.29	-0.3773	19.69	28.38	12.30
site 5	-0.0585	-0.0120	21.15	0.0553	17.01	31.31	13.93
site 5	0.0155	0.0217	18.37	-0.7844	17.15	31.50	16.30
site 5	-0.0017	0.0095	12.35	0.1602	14.50	24.21	12.22
site 5	-0.0713	-0.0171	17.95	-0.5586	16.53	28.80	12.87
site 5	-0.0772	0.0023	18.43	-1.5786	18.34	37.49	15.59
site 5	-0.1096	-0.0072	20.54	-0.1329	18.12	31.52	12.40
site 5	-0.0331	-0.0360	15.48	-0.2918	17.30	26.23	12.40
site 5	-0.0289	0.0023	13.86	1.0794	15.54	27.53	12.91
site 5	0.0641	-0.0229	14.13	-0.4431	16.42	28.09	15.73
site 6	-0.0445	-0.0066	15.76	-0.3578	14.59	28.08	12.87
site 6	-0.1136	0.0051	20.25	0.3508	14.88	34.04	14.03
site 6	-0.0237	-0.0160	13.85	-0.1033	13.05	25.72	12.25
site 6	-0.0403	-0.0154	17.44	0.3858	15.07	29.12	13.36
site 6	-0.0359	-0.0158	20.18	-0.6470	19.53	34.64	14.65
site 6	0.0273	0.0194	15.11	-0.4338	15.58	27.88	14.01
site 6	-0.0483	-0.0028	15.05	-0.2350	15.04	29.70	13.46
site 6	-0.0797	0.0105	19.33	-0.6454	16.00	33.13	14.41
site 6	-0.0288	0.0237	16.03	-0.7291	16.69	31.30	15.26
site 6	-0.0627	0.0243	16.99	-0.3133	17.60	33.71	14.54
site 6	0.0132	-0.0057	19.67	0.0209	19.32	30.43	14.78
site 6	0.0200	-0.0158	14.99	-0.2586	14.77	29.67	15.56
site 6	0.0158	0.0213	17.27	0.4264	14.58	30.16	14.80
site 6	-0.0663	-0.0054	17.25	-0.3075	17.31	30.65	13.65
site 6	-0.0825	-0.0264	16.47	0.2300	16.65	28.64	12.86
site 6	0.0015	-0.0123	11.84	-0.0512	11.01	22.05	10.96
site 6	-0.0557	-0.0135		-0.4006	15.72	31.95	14.14
site 6	0.0070	0.0133	11.97	0.4611	15.04	26.72	13.74
site 6	-0.1285	0.0044	22.19	-0.8279	19.77	36.55	13.63
site 6	-0.1153	-0.0193	21.41	-1.1368	19.06	33.28	12.88
site 6	-0.0520	-0.0211	20.04	-0.5834	18.12	32.87	15.27
site 6	-0.0248	-0.0034	17.75	0.1078	16.91	30.37	14.09
site 6	-0.0420	-0.0306	21.14	-0.0541	18.31	35.67	16.19
site 6	-0.0628	-0.0087	22.37	-0.4194	18.60	35.20	16.45
site 6	-0.0917	0.0351	18.80	-0.1116	17.20	30.57	13.56
site 6	-0.0872	0.0033	20.14	-0.3369	17.46	36.23	15.16
site 6	-0.0744	0.0088	15.20	-0.7891	16.61	34.11	14.21
site 6	-0.1026	0.0193	16.07	-0.5363	18.70	34.24	13.78
site 6	-0.0794	0.0198		-0.4007	16.34	32.83	13.69
site 6	-0.0249	-0.0104	20.62	-0.1331	18.64	34.62	14.97
site 6	-0.0073	-0.0047	18.39	-0.4624	15.60	29.41	15.27
site 6	-0.0317	-0.0226	16.06	0.4449	14.10	26.13	12.73
site 6	-0.0039	0.0121	17.48	-0.3331	17.22	32.56	15.10
site 6	0.0011	-0.0134	16.78	-0.9356	15.73	31.03	14.73
site 6	-0.0670	0.0167	21.66	1.5143	16.84	36.48	17.16
site 6	-0.0488	-0.0163	18.31	-0.2785	15.51	27.84	12.46

site 6	0.0001	0.0120	16.61	-0.1630	16.80	34.34	17.29
site 6	0.0665	-0.0315	14.12	0.0073	13.58	25.20	13.70
site 6	-0.0659	-0.0008	16.73	0.3976	14.82	28.15	11.84
site 6	-0.0313	-0.0376	17.51	-0.4248	15.07	28.31	13.10
site 6	-0.0276	-0.0131	15.61	-0.2702	13.10	27.34	12.85
site 6	-0.0525	-0.0032	15.90	0.1045	15.28	27.28	12.93
site 6	-0.0241	-0.0319	14.86	0.0167	13.70	25.17	12.21
site 6	-0.0735	-0.0120	16.22	1.1850	16.75	27.88	12.14
site 6	0.0097	-0.0164	16.65	-0.1843	16.43	30.05	15.60
site 6	-0.0775	0.0242	20.43	-0.8162	17.41	33.92	14.22
site 6	-0.0404	-0.0016	17.73	0.2432	18.40	34.48	15.18
site 6	-0.0311	-0.0101	17.68	-0.3203	19.48	30.00	14.07
site 6	0.0053	-0.0115	20.15	-0.4625	17.15	31.30	15.26
site 6	-0.0295	0.0129	17.09	0.1386	16.48	30.22	13.37
site 6	-0.0535	0.0014	17.14	-0.8658	16.25	30.76	14.03
site 6	-0.1664	-0.0165	17.35	0.4401	16.06	32.24	10.11
site 6	-0.0125	-0.0242	16.76	0.1111	14.81	28.17	14.03
site 6	-0.0895	-0.0287	19.02	-0.5052	16.05	32.83	13.80
site 6	-0.0235	0.0023	15.09	0.1064	17.10	29.35	13.31
site 6	-0.0345	0.0148	17.30	-0.9280	16.51	29.17	13.83
site 6	0.0135	-0.0012	17.35	-0.1635	15.99	30.93	15.62
site 6	0.0136	-0.0120	17.83	-0.9083	17.34	31.36	15.54
site 6	0.0206	-0.0098	8.30	0.6849	8.56	18.22	8.92
site 6	-0.0132	0.0087	14.48	-0.2809	14.60	29.05	13.59
site 6	-0.1098	-0.0003	20.97	-0.1631	19.57	28.09	13.13
site 6	-0.0202	-0.0009	12.19	-0.6034	14.20	28.07	13.03
site 6	-0.0101	-0.0031	13.66	-0.6301	16.35	27.11	13.08
site 6	0.0300	-0.0191	10.92	0.2635	9.74	19.97	9.68
site 6	-0.0087	-0.0147	11.45	0.1431	9.75	20.28	9.67
site 6	-0.0713	0.0113	21.19	-0.6152	17.69	34.13	14.06
site 6	-0.1590	0.0078	17.29	0.1118	13.03	28.02	9.43
site 6	0.0047	0.0232	12.51	-0.1667	15.30	27.31	12.84
site 6	0.0298	-0.0359	10.85	0.2517	10.45	20.30	11.42
site 6	0.0560	-0.0166	8.85	0.4496	9.59	17.73	9.20
site 6	0.0218	-0.0159	13.90	0.3609	15.23	25.29	13.32
site 6	-0.0348	-0.0040	14.24	-0.5119	16.42	29.49	13.28
site 6	-0.0201	0.0385	16.36	-0.8563	14.97	29.16	14.81
site 6	0.0438	-0.0333	15.67	-0.7066	14.09	29.47	15.30
site 6	-0.0105	0.0265	15.49	-0.5965	14.63	30.33	14.83
site 6	-0.0033	-0.0154	12.89	-0.3662	16.35	26.10	12.80
site 6	-0.0403	0.0092		-0.5234	13.55	25.70	12.20
site 6	-0.0188	-0.0186	14.10	0.6090	14.85	28.04	12.80
site 6	-0.0317	0.0028	17.36	-0.8740	16.46	31.78	15.33
site 6	-0.0883	0.0216	13.70	-0.4512	16.10	30.77	12.44
site 6	-0.0067	-0.0115	14.79	-0.0887	13.15	26.02	13.00
site 6	0.0358	-0.0094	16.68	-0.3623	15.30	28.22	15.07

site 6	-0.0648	0.0154	18.80	-1.3231	17.28	36.90	15.86
site 6	-0.0633	0.0009	19.08	-0.5420	16.19	33.40	14.86
site 7	-0.0500	0.0134	13.96	0.0429	10.79	23.47	10.23
site 7	-0.0239	0.0114		-0.0746	11.86	21.65	10.30
site 7	-0.0580	0.0161	16.23	0.1564	12.63	22.63	10.11
site 7	0.0306	-0.0416	10.89	0.3100	10.46	19.37	10.16
site 7	-0.0570	0.0355	13.06	-0.1672	12.73	23.44	10.14
site 7	-0.0715	0.0227	12.05	-0.0026	11.17	22.89	9.46
site 7	-0.0506	0.0294	12.43	-0.0088	11.99	20.77	9.60
site 7	-0.0922	0.0220	14.67	0.1099	10.51	22.13	9.83
site 7	-0.0539	0.0042		0.2885	11.88	21.46	9.22
site 7	-0.0397	0.0128		0.4383	10.21	18.40	8.20
site 7	-0.0296	-0.0112	12.27	0.6523	9.33	18.41	8.84
site 7	-0.0744	0.0426	11.62	0.3744	11.29	19.18	8.55
site 7	0.0054	0.0029	9.67	0.4806	9.60	17.50	8.77
site 7	-0.1068	0.0087	13.34	0.3656	9.88	20.12	8.36
site 7	-0.0226	0.0104	10.49	0.1775	10.50	20.04	9.54
site 7	-0.0401	0.0002	13.36	0.2690	10.13	20.51	9.45
site 7	-0.0268	0.0116	13.47	0.3103	10.70	20.37	10.14
site 7	-0.0665	-0.0056	12.12	0.2466	10.54	20.05	8.50
site 7	0.0128	-0.0228	11.46	0.4556	9.50	18.60	9.09
site 7	-0.0824	-0.0047	11.42	0.6185	9.12	17.70	7.68
site 7	-0.0084	-0.0196	10.13	0.2774	10.32	19.02	9.03
site 7	-0.0261	-0.0238	12.72	0.2020	10.18	19.90	9.33
site 7	-0.0291	0.0116		0.3306	9.51	19.65	9.20
site 7	0.0053	0.0285	10.59	0.2912	11.02	19.31	9.78
site 7	-0.0370	0.0139	13.07	-0.0627	15.02	20.22	9.00
site 7	-0.0346	-0.0126		-0.3636	12.88	16.92	12.87
site 7	0.0182	0.0075		0.3211	9.94	19.36	9.82
site 7	-0.0873	0.0612	12.80	0.0342	11.42	20.58	9.29
site 7	-0.0191	-0.0008	10.93	0.3231	11.04	18.38	8.50
site 7	-0.0741	-0.0111	10.86	0.2944	9.56	20.31	8.61
site 7	-0.1093	0.0299	14.30	-0.2573	11.61	26.43	11.25
site 7	-0.0114	0.0150	9.79	-0.2685	12.06	22.60	10.77
site 7	-0.0363	0.0164		0.5255	8.83	17.39	7.92
site 7	-0.0592	0.0034	13.70	0.5080	9.83	19.66	8.86
site 7	-0.0223	0.0289		0.2033	9.73	20.19	9.24
site 7	-0.0797	0.0214	10.26	0.1693	10.75	20.53	8.82
site 7	-0.0078	-0.0019	9.80	0.5811	10.73	10.78	9.20
site 7	-0.0730	0.0215		0.3366	9.56	19.46	8.55
site 7	-0.0461	0.0239		-0.3985	11.97	24.20	11.06
site 7	-0.0159	0.0277		0.5211	8.79	17.63	8.33
site 7	0.0313	-0.0215	10.98	0.4261	9.82	18.76	9.73
site 7	-0.0139	0.0041		0.4300	9.80	17.63	8.42
site 7	-0.0655	-0.0022		0.3283	9.57	18.97	8.48
site 7	-0.0028	0.0344		-0.0851	11.50	20.85	10.81

site 7	0.0006	0.0163	12.71	0.1652	9.71	19.39	9.54
site 7	-0.0083	0.0283	11.16	0.3716	9.64	18.48	9.04
site 7	-0.0013	0.0114	10.91	-0.0124	12.20	21.59	10.01
site 7	-0.0366	0.0174		0.3354	10.12	18.76	8.73
site 7	0.0338	-0.0115	8.07	0.1830	11.90	17.61	9.22
site 7	-0.0304	0.0077	11.36	0.0862	11.10	20.62	9.35
site 7	-0.0576	0.0024	13.79	0.0255	11.86	22.20	10.41
site 7	-0.0764	0.0173	12.16	0.0141	11.63	20.90	9.32
site 7	-0.0414	0.0020		0.3145	9.76	19.04	8.28
site 7	-0.0418	0.0174	11.02	0.2571	10.84	19.45	9.42
site 7	-0.0905	0.0358	14.13	0.0116	11.70	20.65	9.10
site 7	-0.0495	0.0209		0.6881	8.68	16.07	7.60
site 7	-0.0197	0.0248		0.1385	10.47	21.08	9.71
site 7	-0.0068	0.0024		0.1589	11.35	18.84	9.03
site 7	0.0078	0.0107		0.3777	11.24	17.00	8.70
site 7	-0.0629	0.0142	9.85	0.6198	9.46	17.08	8.02
site 7	-0.0461	0.0240	14.21	0.0773	11.13	20.52	9.40
site 7	-0.0484	0.0357	12.49	0.2410	11.06	18.82	8.68
site 7	-0.0121	-0.0059		0.4097	9.37	18.08	8.98
site 7	-0.0027	0.0290		0.0961	11.51	19.67	9.80
site 7	-0.0323	0.0008		0.2462	9.67	19.76	9.04
site 7	-0.0354	-0.0051	11.22	0.0510	10.86	20.34	9.52
site 7	-0.0433	-0.0017		0.3031	9.78	19.42	8.45
site 7	-0.0353	-0.0163	9.82	0.4878	9.12	17.82	7.90
site 7	-0.1252	0.0269	11.91	0.2477	10.70	19.35	8.47
site 7	-0.0450	-0.0117	6.90	0.4507	9.60	17.59	8.11
site 7	-0.0437	0.0357		0.4161	9.88	18.50	8.21
site 7	-0.0310	-0.0370	9.14	0.4461	9.08	17.84	8.34
site 7	-0.0589	-0.0014		0.4876	10.04	18.06	7.84
site 7	-0.0210	-0.0149		0.5981	8.91	16.92	8.50
site 7	-0.0008	-0.0033	9.48	0.2931	10.55	17.81	9.38
site 7	-0.0421	0.0500		0.2856	10.50	18.12	8.86
site 7	0.0197	-0.0220	10.20	0.6238	8.82	16.53	8.31
site 7	-0.0264	0.0340		0.6422	8.03	16.66	8.10
site 7	0.0069	0.0066	9.93	0.2705	10.68	18.40	9.23
site 7	-0.0559	0.0215	10.78	0.0280	10.80	20.99	9.64
site 7	-0.0047	0.0106	9.42	0.5220	9.15	17.38	8.55
site 7	-0.0626	0.0104	11.38	0.3319	9.95	18.52	8.50
site 7	-0.0564	-0.0164		0.5110	8.53	18.01	8.11
site 7	-0.0541	0.0373		0.3377	9.36	19.23	8.79
site 8	-0.0172	-0.0188		0.0171	12.72	22.18	10.58
site 8	0.0201	0.0080	13.54	-0.5063	13.62	24.16	12.80
site 8	-0.0510	0.0050	17.69	-0.4904	12.70	27.19	11.72
site 8	0.0037	0.0202	11.47	0.0964	12.27	12.40	11.73
site 8	-0.0238	0.0361	11.95	-0.4389	13.32	24.55	11.87
site 8	0.0513	0.0064	12.06	-0.7792	14.64	26.17	13.93

site 8	-0.0388	-0.0057	17.70	-0.7246	14.17	29.03	12.78
site 8	-0.0228	0.0109	14.37	-0.4667	11.92	25.53	12.07
site 8	0.0066	0.0059	12.46	-0.3226	12.15	23.71	11.84
site 8	0.0100	-0.0407	12.05	0.0347	11.88	22.21	10.95
site 8	0.0320	-0.0026	12.03	-0.4461	13.40	22.26	11.57
site 8	-0.0061	0.0013		-0.2310	12.38	22.90	11.20
site 8	-0.0500	0.0208	30.50	-0.0757	11.85	22.35	9.92
site 8	-0.0462	0.0155	13.89	-0.6417	13.43	26.19	12.39
site 8	-0.0538	-0.0014	12.36	-0.0341	12.00	23.19	10.41
site 8	0.0514	-0.0142	10.88	0.1984	11.53	20.89	11.56
site 8	-0.0341	0.0165	14.34	-0.5957	12.43	26.34	11.84
site 8	-0.1132	0.0295	14.51	-0.0583	12.35	23.31	9.52
site 8	-0.0858	0.0228	18.08	-0.2906	12.39	25.94	11.31
site 8	-0.0352	0.0217	13.35	-0.1773	11.62	23.89	10.78
site 8	-0.0502	-0.0074	14.40	-0.4944	13.34	26.96	12.55
site 8	-0.0573	0.0312	14.43	-0.2917	12.03	23.96	10.96
site 8	-0.0213	0.0010	13.16	0.0941	11.21	23.28	10.82
site 8	-0.0914	-0.0079	14.28	-0.5649	13.49	26.19	11.32
site 8	-0.0405	-0.0220	18.08	-0.7237	13.51	26.91	12.01
site 8	0.0020	-0.0042		-0.1283	12.00	20.61	10.62
site 8	-0.0517	0.0338	22.92	-0.2651	12.96	25.65	11.81
site 8	-0.0191	-0.0168	16.30	-0.3426	12.65	23.47	11.10
site 8	0.0479	-0.0082	12.49	-0.2700	12.56	22.52	11.62
site 8	-0.0260	0.0201	12.32	-0.5014	12.87	24.96	11.90
site 8	-0.0233	-0.0227	13.41	-0.3422	11.30	23.12	12.22
site 8	-0.0308	0.0041	15.09	-0.7553	13.82	26.63	12.42
site 8	0.0396	0.0033	9.64	0.1111	11.07	19.26	9.88
site 8	-0.0768	0.0040	17.26	-0.2627	12.93	26.73	11.45
site 8	-0.0200	0.0163	16.42	-0.6107	13.48	25.66	11.91
site 8	-0.0036	0.0231	7.80	-0.3547	11.72	23.84	11.36
site 8	-0.1079	0.0315	14.74	-0.2717	14.46	25.42	10.19
site 8	0.0147	-0.0179	12.60	-0.4243	13.15	24.43	11.97
site 8	-0.0247	0.0065	11.22	0.0611	11.14	20.79	9.47
site 8	-0.0321	0.0124	12.48	-0.6263	12.64	26.10	12.30
site 8	-0.0860	0.0186	12.95	-0.4154	12.92	25.63	11.21
site 8	-0.0137	0.0088	10.06	-0.0021	11.72	20.86	10.15
site 8	-0.0151	-0.0095	11.93	-0.3434	12.54	24.02	11.35
site 8	-0.0479	0.0215		-0.1042	12.04	22.89	10.98
site 8	-0.0091	0.0135	13.68	-0.5749	14.28	23.39	11.61
site 8	-0.0138	0.0135	10.97	-0.0527	11.43	21.55	10.13
site 8	-0.0336	0.0032	12.47	-0.4607	12.52	24.32	11.34
site 8	-0.0307	0.0091	14.23	-0.3505	13.49	23.67	10.99
site 8	-0.0686	0.0140		-0.6541	14.02	26.33	10.91
site 8	-0.0207	0.0084		-0.3199	11.96	23.15	11.46
site 8	-0.0005	-0.0035		-0.3308	13.40	23.00	11.12
site 8	-0.0384	-0.0147	12.93	-0.2825	11.59	24.65	11.41

site 8	0.0373	0.0267	9.71	-0.0502	12.23	20.00	10.95
site 8	-0.0427	0.0252		-0.2425	12.93	23.89	11.30
site 8	-0.0212	-0.0190	11.62	-0.0291	11.68	21.31	10.72
site 8	-0.0395	-0.0104	13.51	-0.1748	12.25	22.06	10.47
site 8	-0.0235	0.0034	11.69	0.0815	11.29	20.49	9.50
site 8	-0.0791	0.0203	14.19	-0.0948	12.00	23.32	10.35
site 8	-0.0475	0.0375	17.29	-0.3385	12.05	24.53	11.51
site 8	0.0114	0.0000	12.32	0.0728	11.56	21.03	10.57
site 8	-0.0300	0.0017	14.73	-0.3429	13.14	24.90	11.55
site 8	-0.0915	0.0142	15.26	-0.0751	11.95	22.66	10.15
site 8	-0.0135	-0.0056	11.34	-0.0638	11.20	22.78	11.06
site 8	-0.0004	0.0042		-0.6488	13.88	26.20	12.60
site 8	-0.0399	0.0461	14.45	-0.3764	13.29	24.47	11.62
site 8	-0.0524	-0.0002	14.90	-0.1163	12.08	21.84	9.83
site 8	-0.0507	0.0124	14.27	-0.2258	11.84	23.88	10.85
site 8	-0.0124	0.0025	12.39	-0.2812	13.02	22.93	10.95
site 8	-0.0794	0.0127	17.88	-0.6377	15.95	28.55	11.95
site 8	-0.0702	0.0186	16.72	-0.3700	12.97	24.92	11.27
site 8	-0.0494	0.0274	17.06	-0.2066	12.25	25.17	11.74
site 8	-0.0075	0.0135	10.57	-0.3091	11.97	23.12	11.37
site 8	0.0187	-0.0068	9.57	0.1397	10.21	20.33	9.97
site 8	-0.0263	0.0133	18.65	-0.2644	11.36	24.08	11.47
site 8	0.0006	-0.0150	10.90	0.3279	10.30	18.70	9.03
site 8	-0.0064	-0.0256	14.96	0.0170	11.89	20.15	10.50
site 8	-0.0873	0.0100	17.99	-0.1653	13.74	26.88	11.19
site 8	-0.0228	0.0257	13.67	-0.6947	13.39	26.30	12.40
site 8	-0.0962	0.0238	10.67	0.1212	11.12	20.57	8.85
site 8	-0.0292	-0.0092	11.59	-0.1326	12.13	24.07	11.30
site 8	-0.0407	0.0036	13.53	0.0476	11.38	20.66	9.66
site 8	0.0022	-0.0233		-0.2871	12.14	22.44	11.09
site 8	-0.0062	0.0127		-0.2699	12.31	23.16	11.24
site 8	-0.0495	0.0226	11.76	-0.8647	13.16	31.81	12.32
site 9	-0.0450	0.0119	13.13	-0.3568	10.49	25.24	11.32
site 9	-0.0480	0.0451		-0.0731	11.53	20.71	10.15
site 9	-0.0206	-0.0021	12.52	0.2523	10.78	19.37	9.00
site 9	-0.0244	-0.0022	16.64	-0.2696	13.02	26.01	11.70
site 9	-0.0797	0.0028	15.54	0.1542	10.68	21.60	9.24
site 9	0.0026	-0.0144		0.0169	11.35	20.71	10.13
site 9	-0.0679	-0.0123		0.3123	10.85	21.86	9.62
site 9	-0.0570	0.0062	12.54	0.1550	10.60	20.73	9.32
site 9	-0.0540	0.0156	13.31	0.2060	10.74	21.48	9.23
site 9	-0.0077	-0.0124	10.94	0.0756	10.74	20.24	9.83
site 9	-0.0604	0.0114	11.60	0.3715	9.68	18.85	8.41
site 9	0.0027	0.0299	8.64	0.3503	10.16	18.04	9.17
site 9	-0.0936	-0.0100	12.66	0.4537	10.53	18.56	7.95
site 9	-0.0851	0.0083	9.18	0.3667	10.31	19.87	8.18

site 9	-0.0058	-0.0189	11.83	0.5315	11.23	20.83	10.44
site 9	0.0608	0.0010	9.16	0.0988	10.93	18.40	9.92
site 9	-0.0568	-0.0034	13.13	0.0865	11.00	21.23	9.36
site 9	-0.0643	0.0038	13.87	0.3894	11.53	19.78	8.57
site 9	0.0068	0.0134	8.80	0.4140	9.64	17.61	8.79
site 9	-0.0204	-0.0034	8.42	0.4779	9.14	17.42	8.29
site 9	-0.0592	-0.0077	13.24	0.4676	10.21	19.71	8.81
site 9	-0.0668	0.0038	8.75	0.5370	9.01	17.52	8.02
site 9	-0.0342	-0.0118		0.6753	8.80	16.69	7.90
site 9	-0.0153	-0.0160	10.99	0.3250	9.98	17.78	9.10
site 9	-0.0275	0.0247		0.5405	9.38	17.18	8.10
site 9	-0.0395	0.0045	9.65	0.4995	9.25	17.51	9.11
site 9	-0.0376	0.0288	7.87	0.6776	8.72	16.78	8.00
site 9	0.0234	-0.0095	8.53	0.4273	10.32	17.91	9.26
site 9	-0.0334	-0.0030		0.2694	10.95	18.26	8.75
site 9	-0.0753	0.0156	11.66	0.6352	9.17	17.60	7.98
site 9	-0.0266	0.0093		0.4536	9.90	17.62	8.78
site 9	-0.0832	0.0041	11.88	0.6490	9.30	16.74	7.88
site 9	-0.1137	-0.0170	10.91	0.5103	9.98	18.89	7.43
site 9	-0.0231	-0.0082		0.6249	9.30	17.25	8.27
site 9	-0.0574	0.0093	8.51	0.6817	10.05	18.48	8.25
site 9	-0.0129	0.0181	11.16	0.5009	9.87	18.63	8.79
site 9	-0.0181	0.0108	9.44	0.2014	9.81	19.92	9.41
site 9	-0.0579	0.0095	7.52	0.7823	8.52	15.87	7.17
site 9	0.0194	0.0138	9.14	0.6491	8.47	16.87	8.65
site 9	-0.0003	0.0408	9.19	0.3361	10.20	18.13	9.03
site 9	0.0273	0.0271	9.64	0.5302	9.56	16.14	8.35
site 9	-0.0749	0.0176	10.48	0.2433	10.56	19.80	8.62
site 9	-0.0181	-0.0009		0.1520	10.06	20.80	9.43
site 9	0.0145	-0.0074	10.15	0.3689	9.59	18.06	9.38
site 9	-0.0140	0.0205	8.60	0.7013	8.72	16.52	8.00
site 9	-0.0016	0.0171		0.5340	9.32	17.24	8.62
site 9	0.0361	0.0093		0.2872	10.44	18.31	9.50
site 9	0.0162	0.0131	9.18	0.2550	9.57	18.54	9.46
site 9	-0.0439	0.0013	16.30	0.0090	11.75	21.39	10.00
site 9	-0.0646	0.0051	14.99	-0.0336	12.29	21.95	9.68
site 9	0.0289	-0.0209	10.68	0.1859	10.75	18.94	10.04
site 9	-0.0408	-0.0186	9.49	0.5523	9.25	18.01	8.38
site 9	-0.0271	-0.0117	11.00	0.1419	11.15	19.63	9.34
site 9	-0.0397	-0.0271		0.6728	8.82	16.47	7.41
site 9	-0.0404	0.0050		0.7975	8.30	16.72	8.12
site 9	-0.0133	0.0011		0.8730	9.40	15.23	7.50
site 9	-0.0142	0.0227		0.5001	9.35	18.05	8.70
site 9	-0.0325	0.0010		0.1580	11.44	20.44	9.42
site 9	-0.0310	-0.0046		0.5587	9.49	17.20	7.65
site 9	-0.0179	-0.0156	9.73	0.4328	9.66	17.93	8.57

site 9	-0.0433	-0.0195		0.4601	9.57	17.51	8.33
site 9	-0.1251	0.0091	11.62	0.3458	9.91	20.91	8.52
site 9	-0.0174	-0.0217	10.35	0.6173	8.98	16.69	8.08
site 9	-0.0406	0.0022		0.5945	9.43	18.70	8.61
site 9	-0.0575	0.0089	12.42	0.3218	9.75	20.56	9.84
site 9	-0.0291	0.0155	11.65	0.4191	9.79	18.40	8.54
site 9	0.0189	0.0033	10.13	0.4718	9.83	17.64	8.86
site 9	-0.0322	-0.0071		0.6894	9.27	15.70	7.25
site 9	-0.0024	-0.0133		0.1897	10.98	18.82	9.44
site 9	-0.0357	0.0261		-0.0494	11.63	21.59	10.08
site 9	-0.0147	-0.0118	10.60	0.2039	9.94	19.22	9.44
site 9	-0.0399	0.0047	13.62	0.3654	10.70	19.81	9.35
site 9	-0.0640	-0.0006	13.18	0.7488	8.73	16.49	7.24
site 9	-0.0320	0.0124	13.83	0.6194	9.50	17.31	8.24
site 9	-0.0754	-0.0062		0.6255	9.64	18.73	7.77
site 9	-0.0046	-0.0170	9.10	0.4321	9.72	17.52	8.41
site 9	-0.0003	0.0362	9.90	0.3389	9.66	18.41	9.00
site 9	-0.0752	0.0077		0.4188	9.27	19.09	8.20
site 9	0.0678	0.0100	8.41	0.5024	8.96	16.38	8.85
site 9	0.0087	0.0093	9.88	0.3876	10.44	17.40	8.31
site 9	-0.0199	-0.0090		0.7292	8.60	16.22	8.07
site 9	-0.0324	0.0174		0.5357	9.23	17.34	8.04
site 9	-0.0482	-0.0118		0.3432	10.80	19.42	8.75
site 9	0.0196	0.0389		0.5012	9.68	16.70	8.72
